# On-farm dietary supplementation of black seed (*Nigella sativa*) meal in goats: effects on physiological and metabolomic responses during transportation

**DOI:** 10.3389/fvets.2025.1721007

**Published:** 2026-01-22

**Authors:** Priyanka Gurrapu, Phaneendra Batchu, Arshad Shaik, Thomas H. Terrill, Govind Kannan

**Affiliations:** Agricultural Research Station, Fort Valley State University, Fort Valley, GA, United States

**Keywords:** antioxidant activity, black seed meal, catecholamines, metabolomics, stress

## Abstract

Black cumin or black seed (*Nigella sativa*) has many beneficial biological properties, and its processing for oil extraction produces a byproduct known as black seed meal (BSM), which is utilized as an animal feed supplement. An experiment was conducted on a commercial farm to determine the effects of BSM supplementation and long-duration transportation on stress and metabolomic responses and antioxidant and immune capacities in goats. Ninety-six uncastrated male Spanish goats (4–5 months old) were randomly divided into two treatment (TRT) groups. Forty-eight goats were fed a concentrate diet containing 15% BSM, and 48 goats were fed the same diet with no BSM (control, C) in separate corrals for 3 weeks with *ad libitum* water. On the day of the experiment, goats were loaded onto two identical trailers (5 × 2.3 m), with 40 goats/trailer (20 goats/TRT), and were transported for 16 h to simulate a commercial situation. Blood samples were collected at 0 h (15 min after loading), 2 h, 4 h, 10 h, and 16 h of transportation (Time; *n* = 8 goats/Time/TRT) by jugular venipuncture. The dietary BSM supplementation in goats did not affect stress responses, except for tyramine (*p* < 0.05), but Time significantly affected (*p* < 0.05) plasma epinephrine, metanephrine, and normetanephrine. The BSM supplement did not significantly affect the antioxidant and immune status variables. At the metabolome level, 15 amino acids, 4 acylcarnitines, 24 phosphatidylcholines and sphingomyelins, and 13 other metabolites were significantly affected (*p* < 0.05) by TRT. Acylcarnitine (C2), hexadecenoylcarnitine (C16:1), hydroxybutyrylcarnitine (C4OH), β-hydroxybutyric acid, and iso-butyric acid concentrations were higher (*p* < 0.05) in the BSM goats, indicating energy supply was mainly through lipid metabolism. The BSM group had lower (*p* < 0.05) concentrations of glucose, 11 of the amino acids, and TCA cycle metabolites compared to the C group. Supplementation of BSM in the meat goat diet prior to extended road transportation may help them use fat as an energy source instead of breaking down protein. However, at a 15% level, there were no significant effects on antioxidant and immune status indicators determined.

## Introduction

Plant by-product-based feeds are a cost-effective alternative dietary supplement for livestock and have been shown to have several beneficial effects, including improving animal health, welfare, and productivity. Black cumin (*Nigella sativa*) is a small annual herb that is part of the Ranunculaceae family and is used as a source of industrial seed oil and medicine. The presence of alkaloids, coumarins, saponins, flavonoids, terpenes (thymoquinone), fixed oils, and phenolics is responsible for the medicinal activity of *N. sativa*. The active compounds of black cumin seed have been reported to have antioxidant, neuroprotective, anti-inflammatory, immunomodulatory, and analgesic activities, among others (1, 2).

Black cumin seed processing produces a significant amount of byproduct known as black cumin meal (black seed meal, BSM), which may be utilized as an animal feed supplement (3). Due to its high crude protein (330 g/kg), essential amino acids, fat (127 g/kg), and energy contents, BSM is an excellent alternative ruminant feed supplement of high nutritional value (4). Black cumin addition to the diet of goats has been reported to significantly decrease the levels of cortisol, glucose, and total cholesterol during the months of the hot summer season (5). A 10–15% BSM inclusion is generally recommended for small ruminants (6). These authors also noted that BSM does not influence palatability, as an increase in average daily gain was associated with increased dry matter intake. Among other compounds, black cumin contains thymoquinone, a major phytochemical bioactive ingredient (7), which makes up about 30–48% of the volatile oil fraction from black seed (8), although its content in the extract varies depending on the geographical region where the black cumin seeds are produced (9, 10).

Thymoquinone has been demonstrated to modulate the hypothalamic–pituitary–adrenal (HPA) axis and reduce hyperactivity, leading to decreased stress levels in animal models (11). *N. sativa* enhances the scavenging of free radicals, increases activities of superoxide dismutase, glutathione peroxidase, and catalase, and inhibits lipid peroxidation and NF-kB activity, thus imparting a protective effect against oxidative stress (12). This compound inhibits NF-kB by activating the Nrf2/ARE signaling pathway (13). Treatment with black seed and thymoquinone suppresses pro-inflammatory cytokines, such as IL-6 and TNF-alpha, in rats, indicating reduced inflammation and enhanced cell-mediated immunity (8, 14). Thus, thymoquinone can act as an antioxidant agent, inhibiting non-enzymatic peroxidation, increasing immunity, and helping animals tolerate stress (15).

Goats can perform well in places not ideally suited for other livestock and thus are hardy in nature (16). Spanish goats, commonly raised for meat production in the southeastern US, are known to browse a variety of plants and tolerate bitter tastes reasonably well, like most other breeds. Meat goats are frequently moved across vast distances in commercial settings that invariably cause stress and protracted negative aftereffects. During stressful situations, animals experience physiological changes that help them maintain bodily homeostasis; however, when stress becomes too intense and prolonged, these physiological systems are unable to cope, and the negative impacts of stress become obvious. The well-being of animals during transit is becoming an increasingly important societal concern. Antioxidant status, immune function, and physiological and metabolomic responses are all affected by intense stress, such as transportation (17, 18).

Researchers have used numerous physiological markers to assess stress in food animals, and studies have also focused on the accuracy of these animal welfare indices (19). Catecholamines (epinephrine and norepinephrine) and glucocorticoids are thought to form the central components of the endocrine response during stress. Together, these hormones help to orchestrate the body’s response to stress. The plasma levels of catecholamines increase during transportation stress, which may indicate simultaneous activity of both the adrenal cortex and adrenal medulla (20).

Metabolomics, a large-scale study of small molecules, commonly known as metabolites, within cells, biofluids, tissues, or organisms, is emerging as an effective tool to assess animal stress (17, 21, 22). These metabolites can most directly reflect an animal’s physiological status and can be influenced by genetic and environmental factors (22). For example, the plasma metabolome in goats is significantly impacted by stress, with amino acid levels decreasing and medium- and long-chain acylcarnitine concentrations increasing with increasing stress duration (17).

To our knowledge, the information available on the effects of dietary BSM supplementation on the physiological responses to intense and prolonged stress and distress in goats is scant. This study was conducted under commercial conditions to determine the effects of feeding a BSM supplement for 3 weeks before long-duration transportation on stress responses, including antioxidant and immune statuses and metabolomic profiles in goats.

## Materials and methods

### Study design

**Figure 1:**
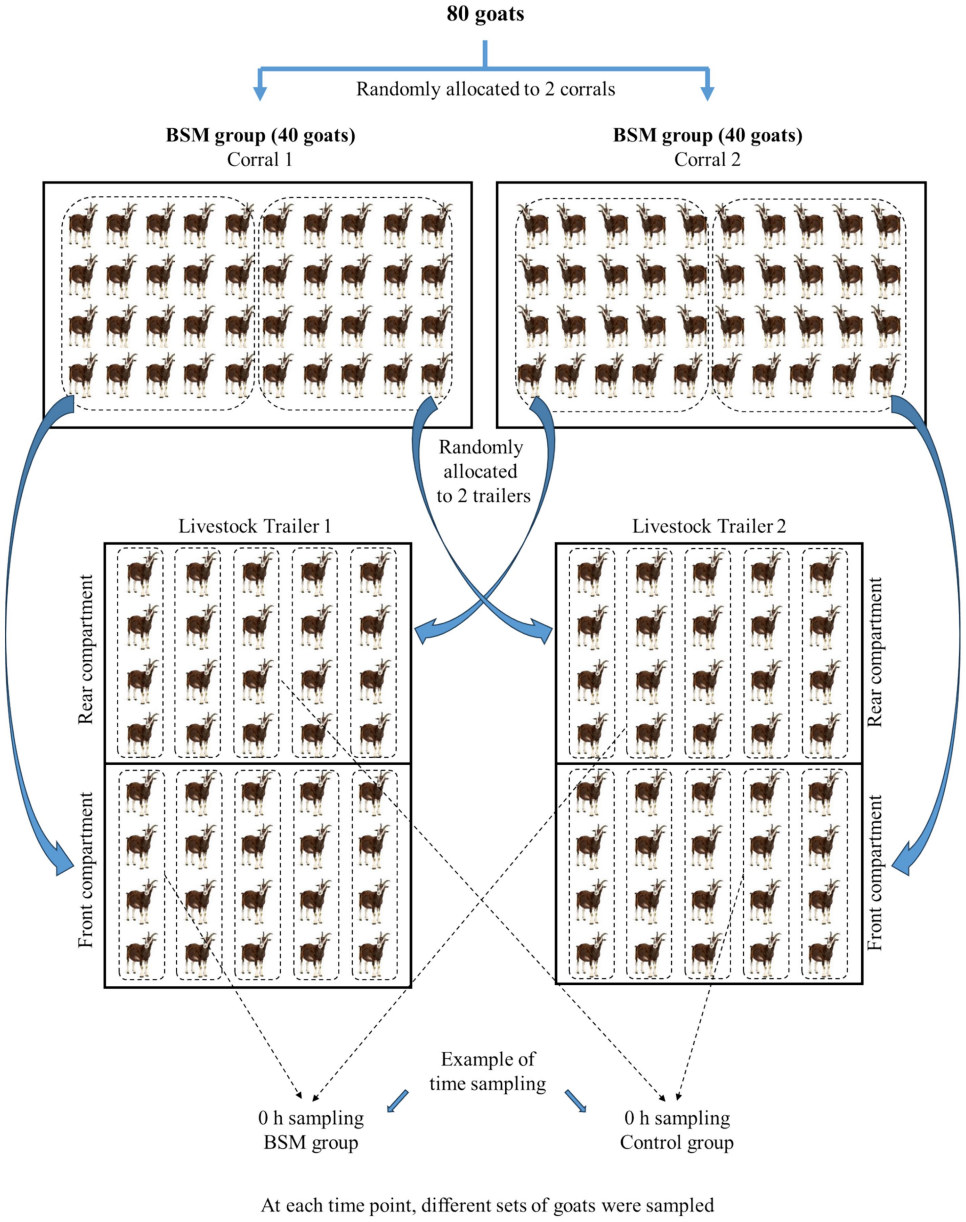
Diagram showing the assignment of goats to treatments (TRT), trailer compartments, and time sampling (time).

Ninety-six uncastrated male Spanish goats (4–5 months old) were used in this experiment conducted at a commercial production facility. Goats were randomly divided into two treatments (TRT) groups and fed either a concentrate diet containing BSM (BSM group; *n* = 48 goats) or a regular concentrate feed mixture with no BSM [control (C) group; *n* = 48 goats] for 3 weeks. The ingredients for both dietary treatments are shown in Table 1. The ingredients were the same for both treatments, but one had BSM included at 15% of the diet, since anecdotal evidence shows that it is a common practice among meat goat producers who have access to this product to include it in the goat feed at 15%. Both TRT groups were kept in separate corrals for 3 weeks with *ad libitum* water and feed (large feeder with adequate feeder space) and shelter. Animals in both treatment groups were deprived of feed overnight prior to transportation. One day prior to transportation, 8 goats from each dietary group were blood sampled to establish baseline values before stress-related changes occurred in the response variables; however, these 16 goats were not transported. On the day of the experiment, goats were loaded onto two identical trailers (5.0 × 2.3 meters) pulled by identical trucks, with 40 goats in each trailer (20 from each TRT), and were transported for 16 h. The sides of the trailers were provided with windows to allow adequate ventilation. The average temperature and humidity measured every 2 h were 26.1 °C and 29.3%, respectively, in trailer 1, and 26.6 °C and 25.6%, respectively, in trailer 2. The goats from the two treatments were kept separate in the two compartments of the trailer (Figure. 1). Blood samples were collected from randomly selected individual animals at 15 min after loading (0 h), 2, 4, 10, and 16 h of transportation (Time; *n* = 8 goats/Time/TRT). Representative samples of the two concentrate feeds were collected and analyzed for nutrient content at a commercial laboratory (Dairy One Forage Laboratory, Ithaca, New York, Table 1).

**Table 1:** Ingredients and chemical composition of experimental diets (Black seed meal, BSM, diet; Control diet, C).

Item	BSM diet	Control diet
Ingredients	%	
Milo	33.15	39.0
Alfalfa pellets	20.4	24.0
Cottonseed hull	12.75	15.0
Cottonseed meal	12.75	15.0
Mineral premix	4.25	5.0
Urea	1.275	1.5
Ammonium chloride	0.425	0.5
Monensin (Rumensin^®^)	0.002	0.002
Black seed meal	15.0	0.0
Proximate composition	%	
DM	89.1	88.7
CP	15.4	15.1
ADF	20.6	22.5
NDF	28.8	30.3
TDN	68.0	67.0

### Blood sampling

For 2, 4, and 10 h sampling, the trucks were stopped for 10 min each time, one experienced animal handler and one trained blood sampler entered the trailer, and a blood sample was collected from each goat by jugular venipuncture into K_2_EDTA-coated vacutainer tubes. The two individuals were deliberate in their approach to avoid any loud noise or rough handling that could agitate the animals. A different set of goats was sampled at each time, and each goat was marked on the horns with a colored marker after sampling to avoid being sampled again. Blood samples were kept on ice until the separation of plasma. The samples were centrifuged (LW Scientific E8 Portafuge, Lawrenceville, GA) immediately at 2,650 × g for 10 min using a portable centrifuge to separate plasma, and the samples were kept frozen on dry ice and transported to the lab. Plasma aliquots were stored at −80 °C for analysis.

All 96 plasma samples from baseline (8/TRT) and experimental animals (40/TRT) were shipped to The Metabolomics Innovation Center (TMIC) at the University of Alberta (Edmonton, Alberta, Canada) for targeted metabolomics and catecholamine analyses. Aliquots of 400 μL of plasma samples were transferred into labeled Eppendorf tubes and then frozen. The Eppendorf tubes were placed in the appropriately labeled cardboard cryo/freezer box, sealed with tape, and then placed in the ziploc bags. The cardboard cryo/freezer box and/or ziploc bag(s) were placed into the styrofoam container. Enough dry ice (at least 2 pounds for every shipping day) was placed in the styrofoam box to keep the samples cold for approximately 48 h. The styrofoam box was sealed, but not taped, to prevent any explosion due to the buildup of CO_2_ gas. The samples and dry ice were prepared and packaged in accordance with the International Air Transport Association or the US equivalent rules.

### Glutathione peroxidase

Plasma glutathione peroxidase (GPx) concentrations were determined using a commercially available Goat GPx ELISA kit (Cat. No. MBS1601455, MyBioSource, Inc., San Diego, CA, United States). Briefly, the plate was precoated with goat GPx antibody, and the sample containing GPx was added, which resulted in the binding of GPX to antibodies coated on the wells. Then, a biotinylated goat GPx antibody was added that bound to GPx in the sample, followed by the addition of Streptavidin-Horseradish Peroxidase (HRP) that bound to the biotinylated GPx antibody. After incubation, the unbound Streptavidin-HRP was washed away during the washing step. A substrate solution was then added, and the color that developed was proportional to the amount of goat GPx. The reaction was terminated by the addition of the acidic stop solution, and the absorbance was measured at 450 nm. The concentrations were determined using a standard curve (1.0–300.0 ng/mL). The sensitivity of the assay was 0.58 ng/mL, and the intra- and inter-assay coefficients of variation were < 8 and <10%, respectively.

### Superoxide dismutase

Plasma superoxide dismutase (SOD) concentrations were determined using a commercially available Goat SOD ELISA kit (Cat. No. MBS268466, MyBioSource, Inc., San Diego, CA, United States). The kit employs the double antibody sandwich technique, which is based on the characteristics of a target analyte with more than two possible epitopes identified by both the pre-coated capture antibody and the detection antibody simultaneously. In this assay, the pre-coated antibody is an anti-goat SOD monoclonal antibody, while the detection antibody is a biotinylated polyclonal antibody. Briefly, the samples and biotinylated antibodies were added to the ELISA plate wells and washed out with buffer solution after respective addition to the wells. Avidin-peroxidase conjugates were then added to the wells, and after thorough washing, TMB substrate was used for color development. The blue coloration, because of peroxidase activity, finally turned yellow after the addition of the stop solution. The color intensity and the quantity of SOD in the sample were positively correlated. The sensitivity of the assay is up to 0.06 ng/mL according to the kit manufacturer.

### Thiobarbituric acid reactive substances

Thiobarbituric acid reactive substances (TBARS) in plasma samples were determined using the Goat TBARS ELISA kit (Cat. No. MBS2614837, MyBioSource, Inc., San Diego, CA, United States). The principle involved in this double antibody sandwich technique is similar to that explained above for the SOD assay.

### Immunoglobulin A

Plasma immunoglobulin A (IgA) was determined using the Goat IgA ELISA kit (Cat. No. MBS734771, MyBioSource, Inc., San Diego, CA, United States), which applies the competitive enzyme immunoassay technique utilizing a polyclonal anti-IgA antibody and an IgA-HRP conjugate. The assay sample and buffer were incubated together with OgA-HRP conjugate in pre-coated plate for 1 h. After the incubation, the wells were decanted and washed five times. The wells were then incubated with a substrate HRP enzyme. The product of the enzyme-substrate reaction formed a blue colored complex. A stop solution was added to stop the reaction, which turned the solution yellow. The intensity of the color was measured spectrophotometrically at 450 nm in a microplate reader (Synergy HTX Microplate Reader, Bio-Tek, Winooski, VT). A standard curve was created relating the intensity of the color to the concentration of the standards, and the IgA concentration in each sample was interpolated from this standard curve. Sensitivity in this assay is 0.1 mg/mL.

### Immunoglobulin G

A commercially available Goat Immunoglobulin G (IgG) ELISA kit (Cat. No. MBS734771, MyBioSource, Inc., San Diego, CA, United States) was used to determine the IgG concentrations in plasma samples. The capture antibody was pre-coated onto 96-well plates, and the biotin-conjugated antibody was used as a detection antibody. The standards, test samples, and biotin-conjugated detection antibody were added to the wells subsequently and washed with wash buffer. The TMB substrates were used to visualize the HRP enzymatic reaction. The TMB was catalyzed by HRP to produce a blue color product that changed to yellow after the addition of the acidic stop solution. The absorbance values were read using a microplate reader, and the unknown concentrations in samples were calculated. The sensitivity of this assay is 0.938 ng/mL.

### Interleukin 6

A Goat Interleukin 6 (IL6) ELISA commercial kit (Cat. No. MBS025544, MyBioSource, Inc., San Diego, CA, United States) was also used for determining plasma IL6 concentrations. The sensitivity of this kit is 2.0 pg./mL, the detection range is 12.5–400.0 pg./mL, and both intra- and inter-assay coefficients of variation are less than 15%.

### Catecholamines and derivatives

Catecholamine profiling was done on plasma samples at TMIC using the method described by Zheng et al. (23). A combined direct injection mass spectrometry (DI-MS) with a reverse-phase mass spectrometry combined with liquid chromatography (LC–MS/MS) custom assay using an ABSciex 4,000 Qtrap (Applied Biosystems/MDS Sciex) tandem mass spectrometry instrument (Applied Biosystems/MDS Analytical Technologies, Foster City, CA, United States) with an Agilent 1,260 series UHPLC system (Agilent Technologies, Palo Alto, CA, United States) was used for analysis of catecholamines and other biogenic amines. The data were analyzed using Analyst 1.6.2.

### Metabolomics

At TMIC, a targeted quantitative metabolomics approach was employed to analyze the samples using a combination of DI-MS with a reverse-phase LC–MS/MS assay. This custom assay, in combination with mass spectrometry, can be used for the targeted identification and quantification of up to 150 different endogenous metabolites, including amino acids, acylcarnitines, biogenic amines and derivatives, uremic toxins, glycerophospholipids, sphingolipids, and sugars (24). The method combines the derivatization and extraction of analytes and the selective mass-spectrometric detection using multiple reaction monitoring (MRM) pairs. The procedure was followed as described in an earlier publication from our lab (21).

### Statistical analysis

In this study, there were only two corrals, one assigned to each of the dietary treatments. Since the corral constitutes the experimental unit for the diet effect, and any effect of TRT cannot be statistically separated from other factors associated with the corral, the results of the diet effect from this exploratory study should be interpreted with caution. For the Time effect, each set of 4 goats that was time-sampled/trailer is considered the experimental unit. The baseline values were not used in the experimental model, but the means and SEM were presented where appropriate. Statistical analysis of antioxidants and immune function data was conducted using mixed procedures in SAS (release 9.4, SAS Institute, Cary, NC, United States) with TRT, Time, and TRT × Time as fixed effects. The data were first examined for normality and homogeneity of variance using the Shapiro–Wilk’s test and Levene’s test, respectively. Log transformation was used when the data did not meet the assumptions of ANOVA; however, they were backtransformed to the original scale before being presented. When significant by ANOVA, the means were separated using the pdiff procedure.

Data from all 80 samples were used for metabolomics and catecholamine statistical analyses. Preprocessing of data and general approach to statistical analysis are similar to those described in Batchu et al. (21). Briefly, since the data for all groups were not normally distributed, Mann–Whitney *U* rank method was used for univariate analysis, and the effect size (Cliff’s Delta method) and fold change (ratio between group medians) were determined. Kruskal–Wallis test was used for one-way ANOVA, and Dunn’s test with Benjamini-Hochberg False Discovery Rate (FDR) correction was used for multiple comparisons. Two-way ANOVA and *post hoc* tests were conducted on log-transformed data, using the Benjamini-Hochberg FDR method to correct *p*-values for multiple comparisons. For all types of comparisons, principal component analysis (PCA) and partial least squares discriminant analysis (PLS-DA) were performed using Metaboanalyst R. Cross-validation and permutation testing were performed for the PLS-DA model. In PLS-DA, Metaboanalyst reports *R*^2^ as *R*^2^*Y* (variance in *Y* explained), *Q*^2^ (cross-validated predictive ability). The accuracy, *R*^2^, and *Q*^2^ of the PLS-DA model were 0.98, 0.92, and 0.80, respectively. The 0.98 value indicates very high prediction accuracy. The high *R*^2^ of 0.92 indicates that the model explains the classes well. An *R*^2^*Y* of 0.92 means the model explains 92% of the variation in the class labels (BSM vs. C). The *Q*^2^ of 0.80 suggests the model is robust and can reliably predict new samples. The *Q*^2^ reflects out-of-sample performance and, together with the permutation test, serves as a key guard against overfitting. The permutation test *p*-value was 0.5e-4, indicating the PLS-DA results were statistically significant. Variable importance in projection (VIP) score plots were then created, with a score of >1.0 indicating that the metabolite is significantly involved in the separation of the classes.

A pathway enrichment analysis was performed using the Metaboanalyst 6.0 platform. The input consisted of a combined list of all metabolites identified as significantly different (*p* < 0.05) between the BSM and C groups. This type of analysis identifies which metabolic pathways are the most over-represented by this list of significant metabolites, highlighting the biological processes most impacted by dietary treatment.

## Results

### Antioxidant status

**Figure 2:**
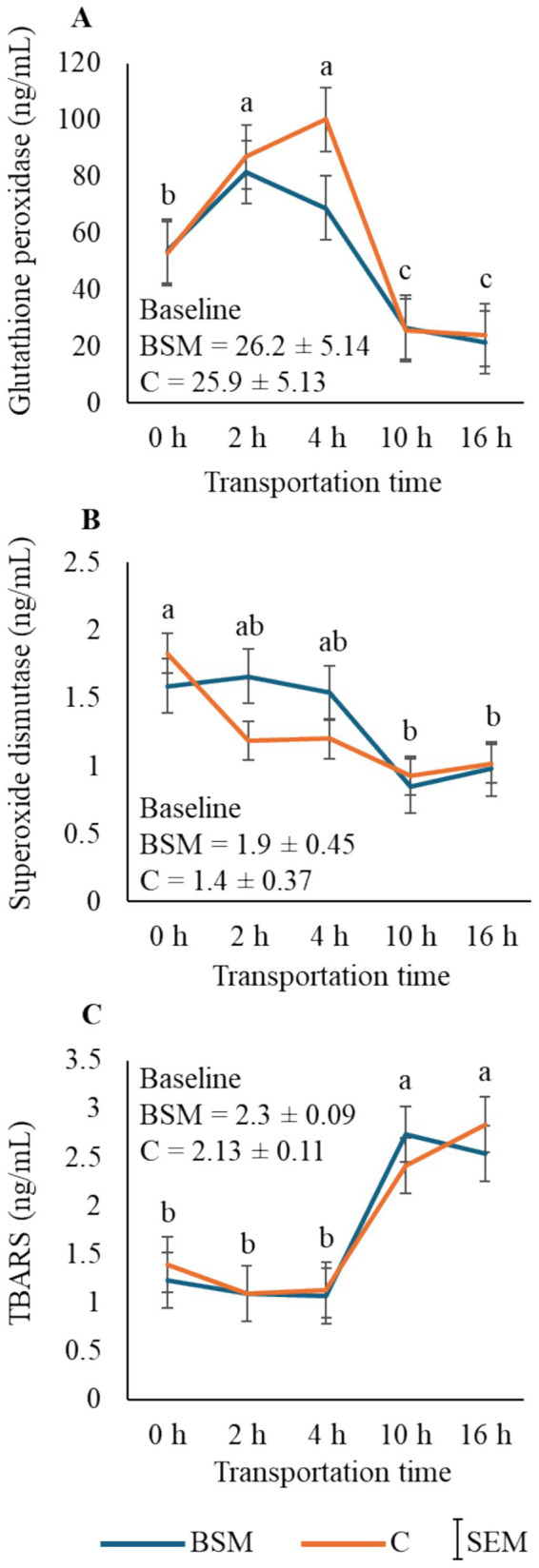
Effects of treatment (BSM = Black seed meal; C = Control) and transportation time on plasma **(A)** glutathione peroxidase, **(B)** superoxide dismutase, and **(C)** thiobarbituric acid reactive substances (TBARS) concentrations in goats. **(A–C)** Time main effect means (*n* = 16 goats/time) with different letters differ significantly by pdiff procedure at *p* < 0.05. Baseline values were not included in the statistical analysis.

Glutathione peroxidase, SOD, and TBARS levels were not affected by TRT or TRT × Time interaction. However, Time had a significant effect on GPx (*p* < 0.01; Figure 2A), SOD (*p* < 0.05; Figure 2B), and TBARS (*p* < 0.01; Figure 2C) concentrations in goats. The mean GPx values increased at 2 and 4 h, reached low levels at 10 h, and remained at that level at 16 h. Plasma SOD concentrations gradually decreased over transportation time and reached low levels at 10 and 16 h. The TBARS values were higher at 10 and 16 h than at 0, 2, and 4 h.

### Immune status

**Figure 3:**
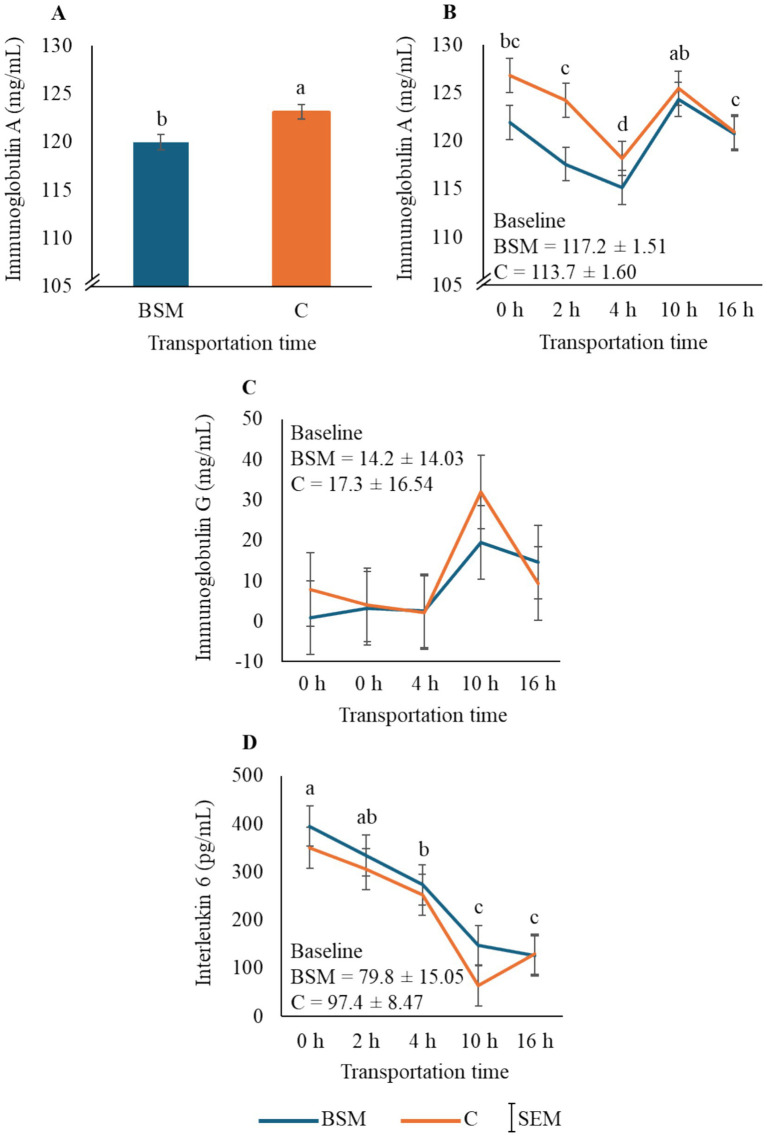
Effects of treatment (BSM = black seed meal; C = control) and transportation time on plasma **(A,B)** immunoglobulin A, **(C)** immunoglobulin G, and **(D)** interleukin 6 concentrations in goats. ^a,b^Bars representing treatment main effect means (*n* = 40 goats/TRT) with different letters are significantly different by PDIFF procedure at *p* < 0.05. ^a,b,c,d^Time main effect means (*n* = 16 goats/Time) with different letters differ significantly by procedure at *p* < 0.05. Baseline values were not included in the statistical analysis.

The overall IgA values were noted to be higher (*p* < 0.01) in the C group than in the BSM group (Figure 3A), primarily due to the differences in values between TRT at 0 and 2 h. Time also had a significant effect (*p* < 0.01; Figure 3B) on plasma IgA; however, there was no clear pattern with the values moving up and down over transportation time. The IgG status was not affected by any of the factors studied (Figure 3C). Interleukin 6 values in both TRT groups decreased over time and reached their lowest values at 10 and 16 h (*p* < 0.01; Figure 3D), while TRT or TRT × Time did not have any significant effect.

### Catecholamines and derivatives

The effects of TRT on the concentrations of catecholamines and their derivatives are depicted in a heatmap (Supplementary Figure 1) created using normalized concentration ranges (from 0 to 1). High, medium, and low points on the color bar correspond to 0.95, 0.5, and 0.05, respectively. Tyramine concentration was observed to be higher (*p* < 0.05) in the C group compared to the BSM group of goats (Supplementary Figure 2). The BSM treatment did not have any significant effect on dopamine, phenylethylamine, 5-methoxytryptamine, epinephrine, norepinephrine, metanephrine, and normetanephrine concentrations.

**Figure 4:**
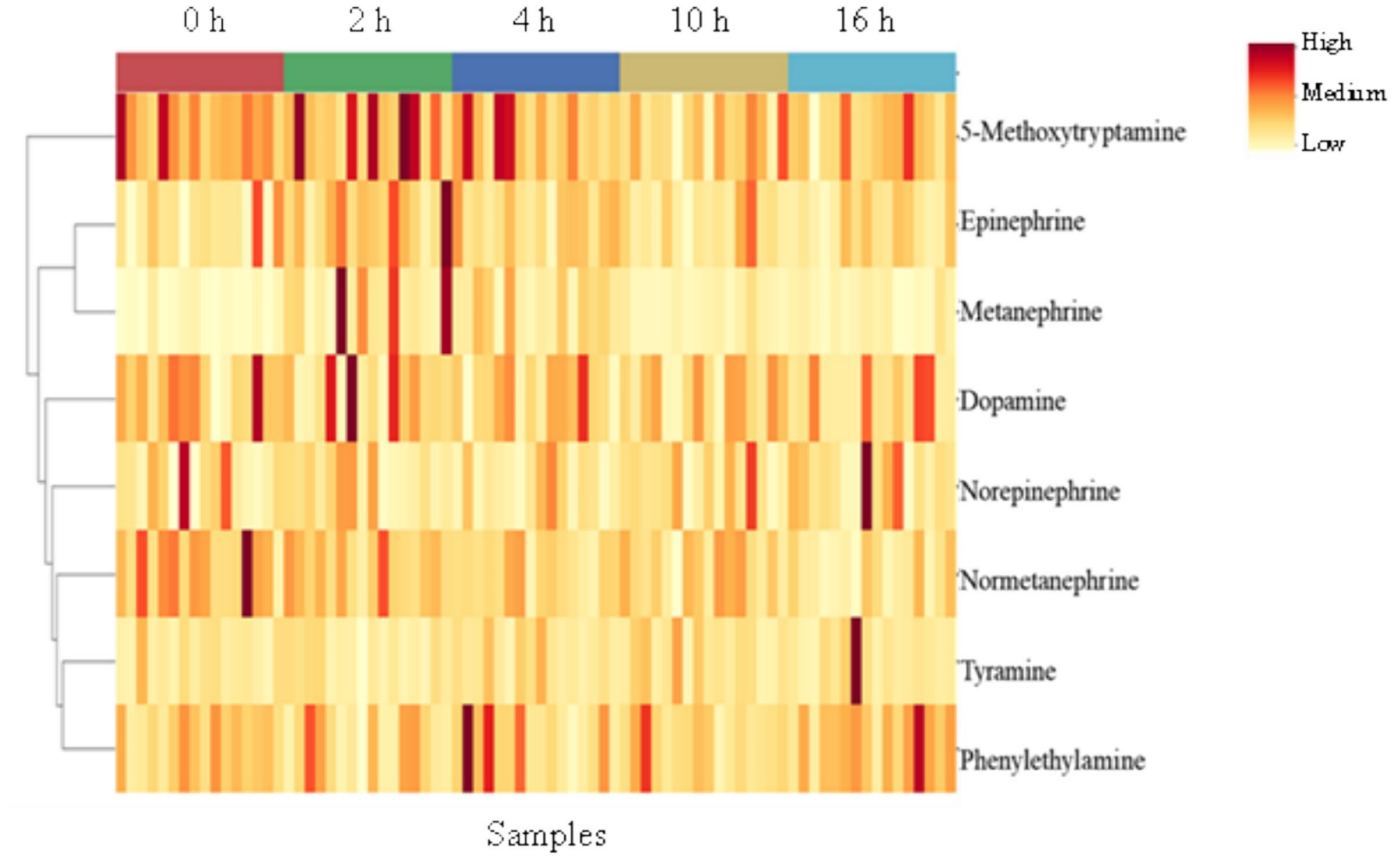
Heatmap of plasma catecholamines and derivatives clustered by transportation time.

Time had a significant effect (*p* < 0.05) on metanephrine, normetanephrine, and epinephrine concentrations (Supplementary Figure 3). The concentration of normetanephrine decreased with increasing transportation time, while the concentrations of epinephrine and metanephrine increased during the first 4 h of transportation and then decreased. The effects of transportation time on the concentrations of catecholamines and their derivatives are shown using a heatmap (Figure 4). The interaction effects were not significant.

### Metabolomics

Involvement in biological processes related to antioxidant capacity, immune function, and/or energy metabolism was used as the primary metabolite selection criterion in this study. Intense stress in goats has been shown to negatively affect these biological processes, while thymoquinone, present in black seed, has been reported to help animals combat these negative effects.

At the metabolome level, 15 amino acids, 4 acylcarnitines, 24 phosphatidylcholines and sphingomyelins, and 13 other metabolites were affected (*p* < 0.05) by TRT. Of the significantly affected amino acids, 11 (phenylalanine, tyrosine, tryptophan, alanine, glutamine, methionine, proline, glutamic acid, taurine, threonine, and asparagine) were higher (*p* < 0.05) in the C group compared to BSM group, while isoleucine, valine, ornithine, and methyl histidine were lower (*p* < 0.05) in the C group compared to the BSM group (Table 2 and Supplementary Figure 4). The concentrations of three of the acylcarnitines [acetylcarnitine (C2), hexadecenoylcarnitine (C16:1), and hydroxybutyrylcarnitine (C4OH)] were lower (*p* < 0.05) in the C group than the BSM group, while the propionylcarnitine (C3) concentration was higher (*p* < 0.05) in the C group compared to the BSM group (Table 3 and Supplementary Figure 5). In this study, treatment appeared to have effects (*p* < 0.05) on 15 phosphatidylcholines and 9 sphingomyelins (Table 4 and Supplementary Figure 6), in addition to several other metabolites (Table 5 and Supplementary Figure 7).

**Table 2:** Amino acids significantly (*P* < 0.05) affected by treatment (BSM = Black seed meal; C = Control; *n* = 40 goats/TRT) in goats.

Metabolite	*P*-value	FDR^1^	Fold change	Effect size	Effect level	Direction of change
Phenylalanine	1.30 × 10^−6^	1.60 × 10^−5^	1.23	−0.61	Large	↑ in C
Tyrosine	7.45 × 10^−6^	6.05 × 10^−5^	1.54	−0.56	Large	↑ in C
Tryptophan	7.95 × 10^−6^	6.05 × 10^−5^	1.43	−0.56	Large	↑ in C
Alanine	1.90 × 10^−5^	1.11 × 10^−4^	1.31	−0.54	Large	↑ in C
Glutamine	7.47 × 10^−4^	2.55 × 10^−3^	1.17	−0.41	Medium	↑ in C
Methionine	2.20 × 10^−3^	6.39 × 10^−3^	1.20	−0.37	Medium	↑ in C
Proline	7.16 × 10^−3^	0.018	1.21	−0.32	Small	↑ in C
Glutamic acid	0.011	0.026	1.05	−0.30	Small	↑ in C
Taurine	0.011	0.026	1.52	−0.30	Small	↑ in C
Threonine	0.016	0.035	1.22	−0.28	Small	↑ in C
Isoleucine	0.018	0.038	0.88	0.27	Small	↓ in C
Valine	0.021	0.044	0.89	0.26	Small	↓ in C
Ornithine	0.025	0.050	0.87	0.26	Small	↓ in C
Asparagine	0.034	0.061	1.33	−0.24	Small	↑ in C
Methyl histidine	0.048	0.086	0.94	0.22	Small	↓ in C

^1^False discovery rate.

**Table 3:** Acylcarnitines significantly (*P* < 0.05) affected by treatment (BSM = Black seed meal; C = Control; *n* = 40 goats/TRT) in goats.

Metabolite	*P*-value	FDR^1^	Fold change	Effect size	Effect level	Direction of change
Acetylcarnitine (C2)	2.25 × 10^−9^	5.56 × 10^−8^	0.42	0.76	Large	↓ in C
Hexadecenoylcarnitine (C16:1)	1.79 × 10^−5^	1.11 × 10^−4^	0.89	0.54	Large	↓ in C
Propionylcarnitine (C3)	5.77 × 10^−3^	0.015	1.25	−0.33	Small	↑ in C
Hydroxybutyrylcarnitine (C4OH)	0.017	0.037	0.74	0.28	Small	↓ in C

^1^FDR = False discovery rate.

**Table 4:** Phosphatidylcholines and sphingomyelins significantly (*p* < 0.05) affected by treatment (BSM = Black seed meal; C = Control; *n* = 40 goats/TRT) in goats.

Metabolite	*P*-value	FDR^1^	Fold change	Effect size	Effect level	Direction of change
Acyl alkyl phosphatidylcholine C36:0	2.91 × 10^−11^	2.88 × 10^−9^	2.06	−0.85	Large	↑ in C
Lysophosphatidylcholine C18:2	1.51 × 10^−10^	7.47 × 10^−9^	0.43	0.82	Large	↓ in C
Hydroxysphingomyelin C22:2	1.89 × 10^−9^	5.56 × 10^−8^	1.33	−0.77	Large	↑ in C
Lysophosphatidylcholine C17:0	9.84 × 10^−9^	1.95 × 10^−7^	1.72	−0.73	Large	↑ in C
Sphingomyelin C20:2	3.03 × 10^−7^	5.00 × 10^−6^	0.70	0.65	Large	↓ in C
Lysophosphatidylcholine C20:4	6.98 × 10^−7^	9.87 × 10^−6^	0.67	0.63	Large	↓ in C
Diacylphosphatidylcholine C32:2	1.64 × 10^−6^	1.78 × 10^−5^	0.78	0.60	Large	↓ in C
Sphingomyelin C18:1	1.08 × 10^−5^	7.61 × 10^−5^	0.73	0.55	Large	↓ in C
Hydroxysphingomyelin C24:1	2.50 × 10^−5^	1.37 × 10^−4^	1.24	−0.53	Large	↑ in C
Lysophosphatidylcholine C18:0	1.04 × 10^−4^	5.14 × 10^−4^	0.83	0.48	Large	↓ in C
Diacylphosphatidylcholine C40:1	1.16 × 10^−4^	5.48 × 10^−4^	1.13	−0.48	Large	↑ in C
Diacylphosphatidylcholine C38:6	2.35 × 10^−4^	1.06 × 10^−3^	0.72	0.46	Medium	↓ in C
Hydroxysphingomyelin C22:1	2.61 × 10^−4^	1.08 × 10^−3^	1.17	−0.45	Medium	↑ in C
Lysophosphatidylcholine C14:0	6.66 × 10^−4^	2.44 × 10^−3^	1.21	−0.42	Medium	↑ in C
Hydroxysphingomyelin C16:1	7.12 × 10^−4^	2.52 × 10^−3^	1.14	−0.41	Medium	↑ in C
Diacylphosphatidylcholine C40:2	8.40 × 10^−4^	2.77 × 10^−3^	1.14	−0.41	Medium	↑ in C
Sphingomyelin C16:1	9.57 × 10^−4^	3.06 × 10^−3^	0.92	0.40	Medium	↓ in C
Sphingomyelin C16:0	1.36 × 10^−3^	4.21 × 10^−3^	0.88	0.39	Medium	↓ in C
Diacylphosphatidylcholine C40:6	1.59 × 10^−3^	4.78 × 10^−3^	0.83	0.38	Medium	↓ in C
Lysophosphatidylcholine C18:1	3.28 × 10^−3^	8.78 × 10^−3^	0.82	0.35	Medium	↓ in C
Diacylphosphatidylcholine C38:0	8.18 × 10^−3^	0.020	0.88	0.31	Small	↓ in C
Alkylacyl phosphatidylcholine C40:6	0.014	0.031	1.10	−0.29	Small	↑ in C
Sphingomyelin C18:0	0.025	0.050	0.89	0.26	Small	↓ in C
Diacylphosphatidylcholine C36:0	0.033	0.061	1.10	−0.24	Small	↑ in C

^1^FDR = False discovery rate.

**Table 5:** Other metabolites significantly (*P* < 0.05) affected by treatment (BSM = Black seed meal; C = Control; *n* = 40 goats/TRT) in goats.

Metabolite	*P*-value	FDR^1^	Fold change	Effect size	Effect level	Direction of change
Propionic acid	1.80 × 10^−6^	1.78 × 10^−5^	1.22	−0.60	Large	↑ in C
β-Hydroxybutyric acid	6.82 × 10^−6^	6.05 × 10^−5^	0.54	0.57	Large	↓ in C
Carnosine	1.45 × 10^−5^	9.57 × 10^−5^	1.40	−0.54	Large	↑ in C
Methylmalonic acid	6.54 × 10^−5^	3.41 × 10^−4^	1.93	−0.50	Large	↑ in C
Isobutyric acid	2.46 × 10^−4^	1.06 × 10^−3^	0.87	0.45	Medium	↓ in C
α-Ketoglutaric acid	2.95 × 10^−4^	1.17 × 10^−3^	1.16	−0.45	Medium	↑ in C
Pyruvic acid	5.72 × 10^−4^	2.18 × 10^−3^	1.18	−0.42	Medium	↑ in C
Fumaric acid	2.48 × 10^−3^	7.01 × 10^−3^	1.20	−0.37	Medium	↑ in C
Uric acid	2.92 × 10^−3^	8.02 × 10^−3^	1.24	−0.36	Medium	↑ in C
Betaine	0.011	0.026	1.21	−0.30	Small	↑ in C
Glucose	0.027	0.053	1.16	−0.25	Small	↑ in C
Succinic acid	0.028	0.053	1.12	−0.25	Small	↑ in C
Trimethylamine N-oxide	0.034	0.061	1.53	−0.24	Small	↑ in C

^1^FDR = False discovery rate.

**Figure 5:**
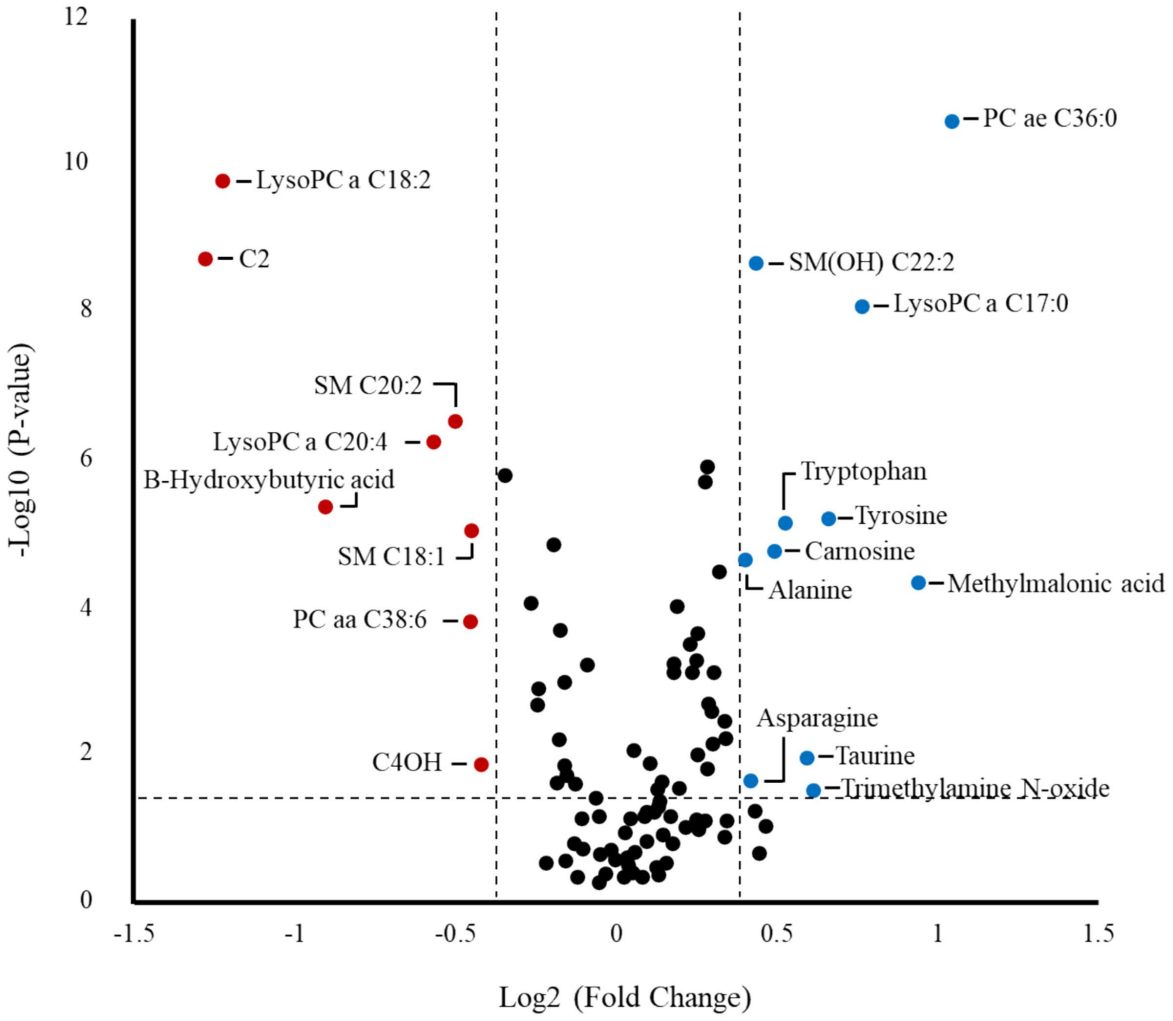
Volcano plot showing metabolites significantly affected by dietary treatment. The names of metabolites with *p*-values below 0.05 and fold change < 0.77 or > 1.3 are shown within the plot, and the absence of metabolite names indicates that none of the metabolites satisfied these criteria.

**Figure 6:**
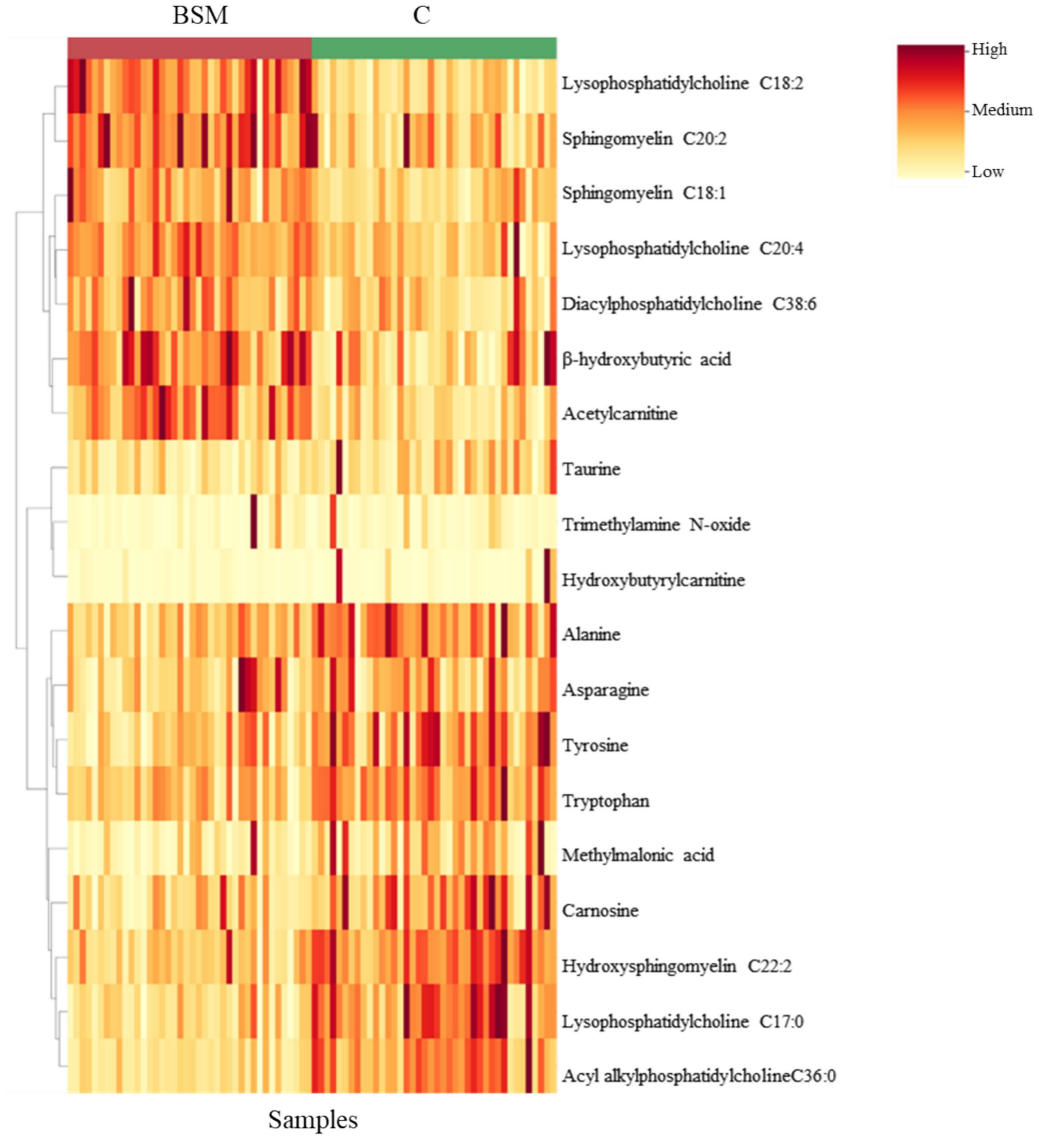
Heatmap of significantly affected (*p* < 0.05) plasma metabolites clustered by treatment.

**Figure 7:**
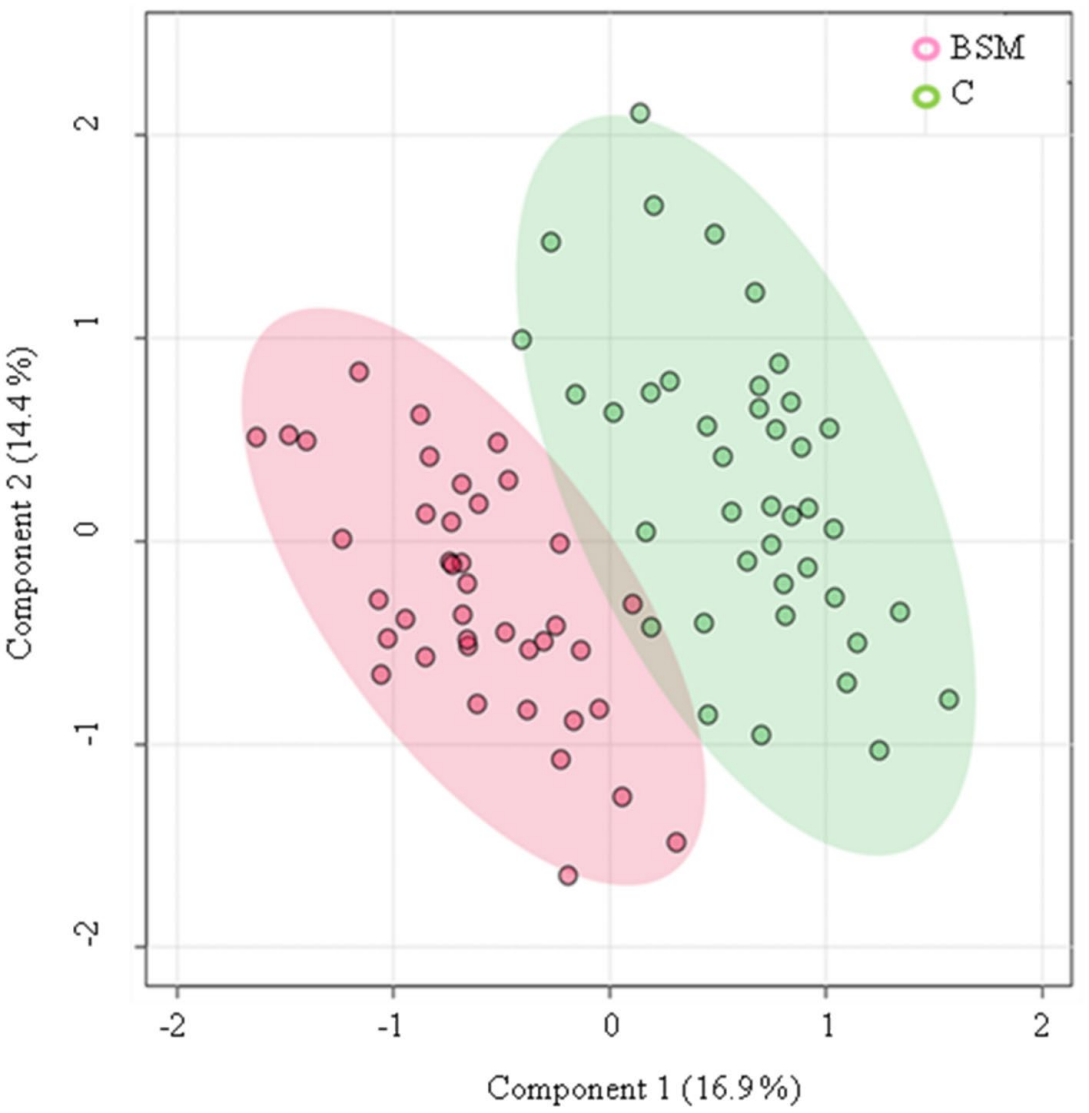
PLS-DA plot of principal components 1 and 2 for treatment classes (BSM = Black seed meal, C = Control; *p* < 0.05) in goats.

**Figure 8:**
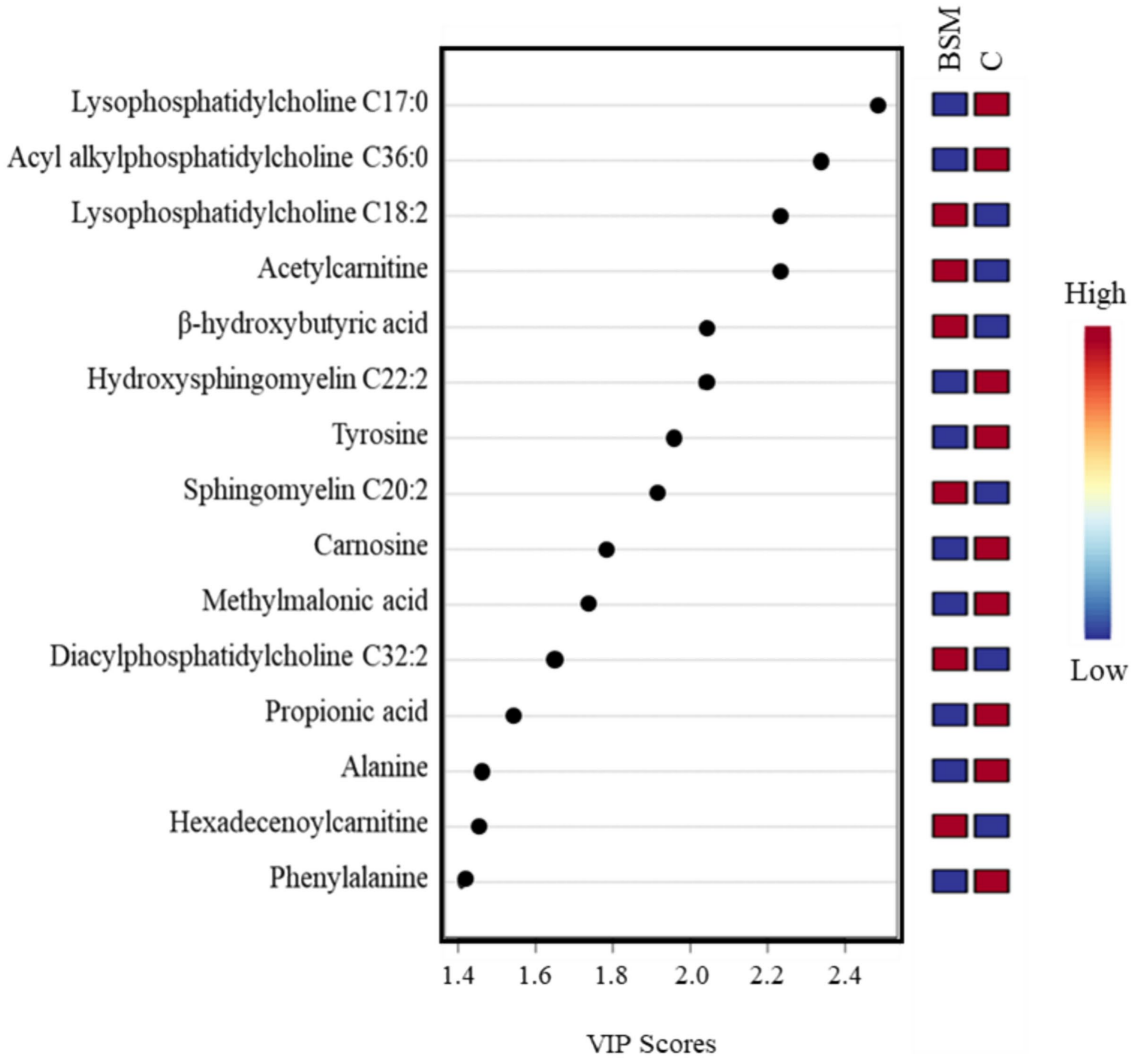
PLS-DA VIP plot showing the metabolites (VIP scores > 1.5) that significantly contribute to the differences between treatment (BSM = Black seed meal, C = Control) groups in goats. The metabolite concentrations averaged across all time points were used in PLS-DA model (*p* < 0.05).

A volcano plot identified that 19 metabolites were affected by TRT (Figure 5). The relative abundance of metabolites affected by TRT was visualized by means of a heat map (Figure 6). The PCA plot created to separate metabolites by TRT (by principal components 1 and 2) revealed partial clustering of the BSM and C groups (Supplementary Figure 8). The total variance of the principal components for the two groups contributed to 33.3% of the PCA model (PC 1 = 21.9%, PC 2 = 11.4%). To maximize the separation of the groups observed by PCA, a PLS-DA was applied. In the PLS-DA model for the two groups, the two components contributed to a total variance of 31.3% (Component 1 = 16.9%, Component 2 = 14.4%). The two groups showed minimal overlapping, with the cluster larger in C compared to the BSM group (Figure 7). When averaged across all time points, lysophosphatidylcholine C17:0, acyl-alkyl-phosphatidylcholine C36:0, lysophosphatidylcholine C18:2, acetylcarnitine, β-hydroxybutyric acid, hydroxysphingomyelin C22:2, tyrosine, sphingomyelin C20:2, carnosine, methylmalonic acid, diacyl-phosphatidylcholine C32:2, propionic acid, alanine, hexadecenoylcarnitine, and phenylalanine were the top 15 metabolites identified by the PLS-DA multivariate model (*p* < 0.05) and VIP values as having the greatest influence (VIP scores >1.5) in separating the BSM and C group (Figure 8).

Time had a significant effect (*p* < 0.05) on 18 amino acids (Supplementary Figure 9), 5 acylcarnitine (Supplementary Figure 10), and 7 phosphatidylcholines and sphingomyelins (Supplementary Figure 11). In addition, several other metabolites were also significantly affected by Time (*p* < 0.05; Supplementary Figure 12). The relative abundance of metabolites significantly affected by Time was visualized by means of a heat map (Supplementary Figure 13). The PLS-DA plot showed a total variance of 28.8% (Component 1 = 13.2%, Component 2 = 15.6%), as shown in Supplementary Figure 14.

**Figure 9:**
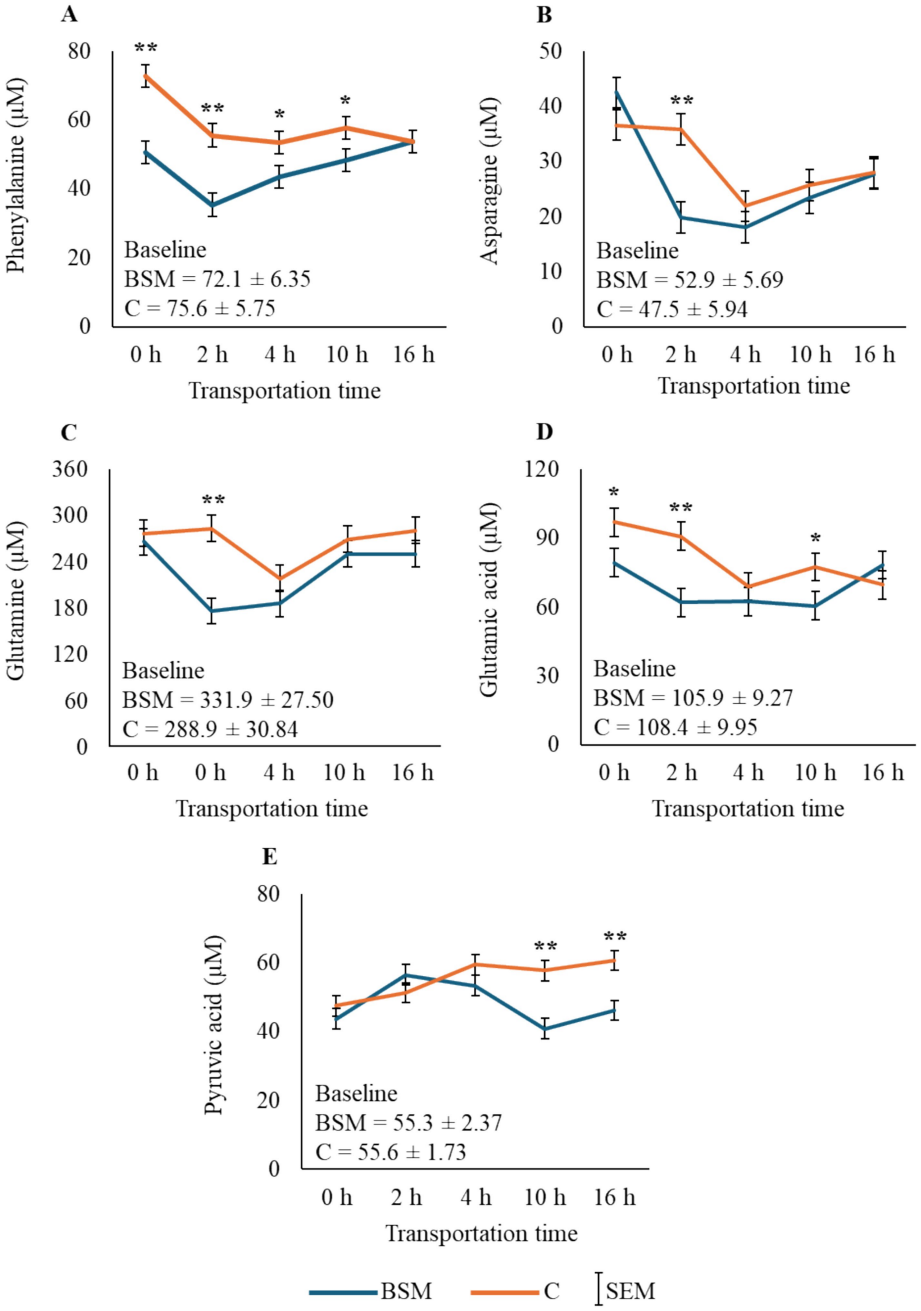
Examples of amino acids (**A** phenylalanine, **B** asparagine, **C** glutamine, **D** glutamic acid) and pyruvic acid **(E)** showing significant TRT × time effect (*p* < 0.05; *n* = 8 goats/time/TRT). Treatment means are different at time points with asterisk(s) (*p < 0.05, **p < 0.01). Baseline values were not included in the statistical analysis.

Interaction effects were significant (*p* < 0.05) for pyruvic acid and eight amino acids (phenylalanine, tyrosine, tryptophan, glutamine, methionine, threonine, asparagine, and glutamic acid). Examples of amino acids with significant TRT × Time effects, along with pyruvic acid, are shown in Figure 9. The interaction effect was also significant (*p* < 0.05; Supplementary Figure 15) for one acylcarnitine [hexadecanoylcarnitine (C16:1)] and one phosphatidylcholine (lysophosphatidylcholine C17:0). The differences in energy-sourcing strategies in BSM and C groups are presented in the form of a mechanistic diagram in Supplementary Figure 16.

**Figure 10:**
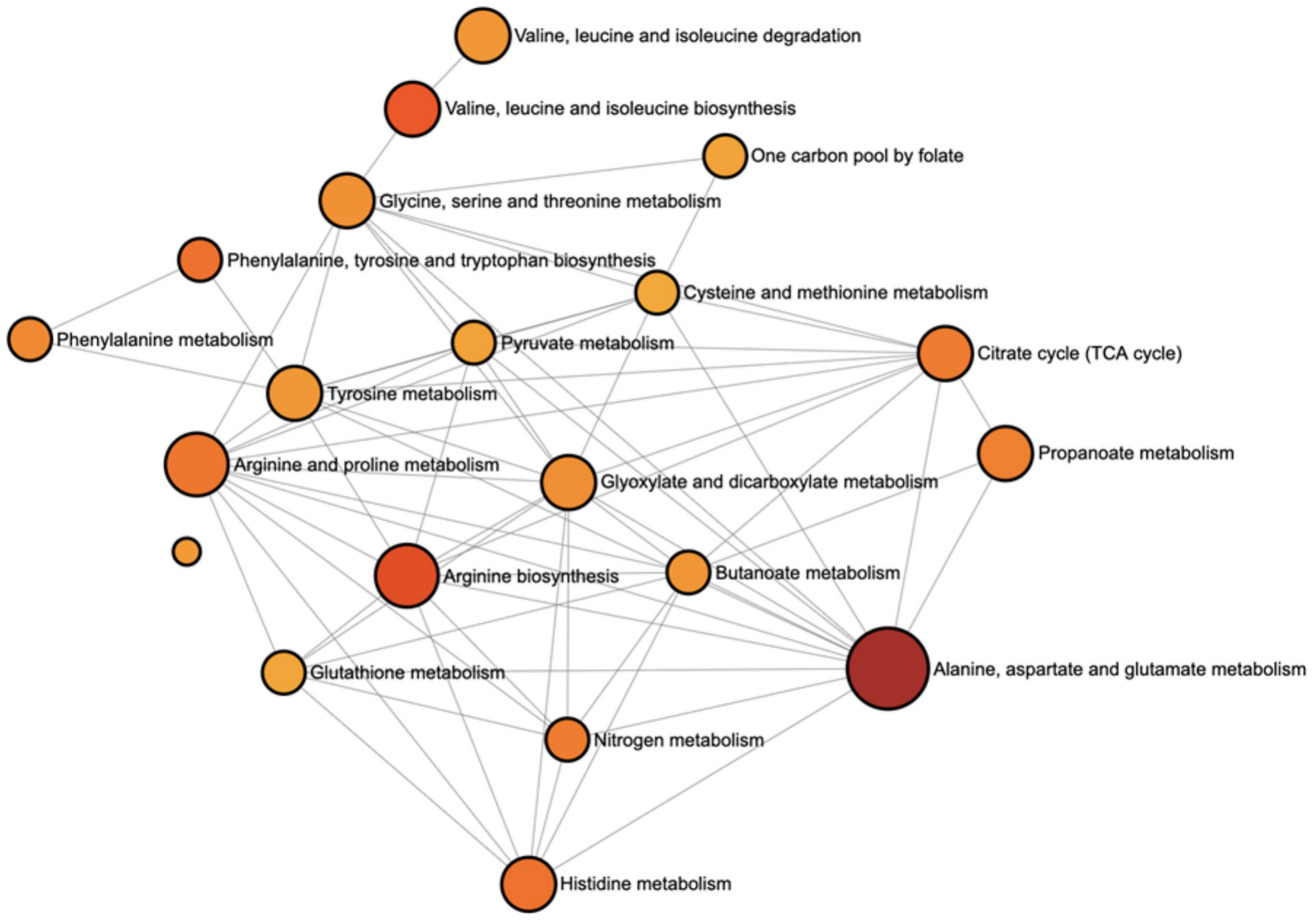
Results of the pathway analysis conducted using a list of all metabolites identified as significantly different (*p* < 0.05) between the BSM and C groups.

The pathway analysis identified that the most significantly impacted pathways were overwhelmingly related to amino acid metabolism and central energy production. The most prominent pathways included (i) the alanine, aspartate, and glutamate metabolism, (ii) the arginine and proline metabolism, (iii) the valine, leucine, and isoleucine degradation, and (iv) the phenylalanine metabolism. The tricarboxylic acid cycle (TCA cycle) was also identified as a highly significant pathway (Figure 10).

## Discussion

In the current study, GPx and SOD concentrations and TBARS values were not differentially influenced by TRT, and the time patterns in both TRT groups were similar. These patterns may suggest that transportation caused extreme stress, which could have overridden the differential effects of dietary treatment. Among other compounds, black cumin contains thymoquinone, which has been reported to act as an antioxidant agent, as it inhibits the non-enzymatic peroxidation, increases immunity, and helps animals in tolerating heat stress (15). The effect noted in our study was the reverse of what has been reported in the literature. Furthermore, IgA levels were higher in the C group compared to the BSM group in the present study. *Nigella sativa* extracts at high concentrations have been reported to suppress lymphocyte response to mitogens and phagocytic activity of polymorphonuclear leukocytes *in vitro* (25). Thymoquinone has been reported to have higher antioxidant activity at lower levels and has a prooxidant effect at higher levels (26), which may suggest that the inclusion of 15% BSM in goat feed, as practiced by meat goat producers, could be higher than optimum. Similar products have been tested in other countries in livestock and poultry. For example, Ramdani et al. (6) studied the effect of 5, 10, and 15% inclusion levels in lambs and observed that approximately 10% BSM in the diet is optimum for increasing average daily gain, although the optimal level of BSM in small ruminants that results in the most favorable effect on antioxidant and immune capacities is not known. The effects of BSM on antioxidant and immune status indicators need further investigation, particularly to determine an ideal level for inclusion in the diet that could help animals combat intense stress conditions.

The elevated epinephrine and metanephrine concentrations at 2 and 4 h of transportation and their gradual decline thereafter were consistent with previous findings in goats, for example, by Nwe et al. (27) and Kannan et al. (28). This reduction in blood concentrations of these catecholamines is due to the decrease in fear and emotional stress caused by the initial novelty of the environment and experience, which also reflects variations in the activity of the sympatho-adrenal medullary axis over time during transportation in goats (27). Metanephrine and normetanephrine are O-methylated metabolites of catecholamines, epinephrine and norepinephrine, respectively, and are reliable indicators of stress under practical circumstances (28). Among the 8 catecholamines analyzed, tyramine concentrations were significantly affected by TRT. The BSM group had lower concentrations than the C group. Tyramine is a naturally occurring trace amine that acts as a catecholamine-releasing agent. As an indirect sympathomimetic agent, it releases norepinephrine from sympathetic nerve terminals (29). Tyramine is part of a stress response, and its concentration increases when an animal encounters stress. In goats, a previous experiment showed that tyramine concentrations increased with increasing levels of stress (30). The lower mean tyramine concentrations in the BSM goats compared to the C goats may indicate that BSM supplementation likely reduced plasma tyramine concentrations, thus indicating a lower stress response.

Phenylalanine or tyrosine serves as the precursor to catecholamines dopamine, norepinephrine, and epinephrine (31, 32). Tyrosine is also converted to tyramine via decarboxylation catalyzed by the enzyme tyrosine decarboxylase (33). In our study, the levels of amino acids phenylalanine and tyrosine, along with 9 other amino acids, were lower in the BSM group compared to the C group of goats, which may also explain the lower tyramine concentrations in the BSM group. Overall, BSM had no significant effect on catecholamine responses, except for tyramine, while the responses over time during transportation confirmed the effects noted in previously reported studies.

The primary effect that became apparent from metabolomics analysis was the change in energy-sourcing strategies used by the two groups of goats when exposed to transportation stress. Lipid metabolism predominated in the BSM goats, and protein catabolism was pronounced in the C goats, resulting in elevated amino acid concentrations in the latter group (Supplementary Figure 16). The higher concentrations of phenylalanine, tyrosine, tryptophan, glutamine, methionine, threonine, asparagine, and glutamic acid in the C group than the BSM group during the first 4 h of transportation, and the absence of differences between the two groups as the journey prolonged, resulted in significant TRT × Time interaction effects. Not only does this time pattern indicate that the BSM diet reduced stress during the first 4 h of transportation, but it also suggests that in the C goats, there was increased mobilization of amino acids from muscles for gluconeogenesis. The higher pyruvic acid concentrations in the C group compared to the BSM group at 10 h and 16 h of transportation, noticed in our study, further confirm that the amino acids are being used for glucose production, as these are both glucogenic and ketogenic (phenylalanine, isoleucine, threonine, tryptophan, and tyrosine) in function (34). The increase in some of these amino acids noticed in the C group could also be due to a higher need for neurotransmitter production during stress. The overall concentrations of 11 amino acids in the BSM group were lower compared to the C group, despite the fact that black cumin seeds contain high amounts of essential amino acids (9). This may suggest that short-term dietary BSM supplementation to goats may have the beneficial effect of lowering the stress response and resultant protein catabolism during long-duration transportation, as shown by lower plasma glucose concentration in the BSM group.

Transportation and treatment had a significant interaction effect for hexadecanoylcarnitine (C16:1), which has been associated with fasting and ketosis, among other conditions (35). Acetylcarnitine (C2), hexadecenoylcarnitine (C16:1), and hydroxybutyrylcarnitine (C4OH) were higher in the BSM group compared to the C group. The surge in the end products of short-chain acylcarnitine (C4OH) correlates with the more commonly measured ketone species β-hydroxybutyrate (36). The β-hydroxybutyrate levels were higher in the BSM group in our study. The rise in ketone levels supports the idea that there has been a switch from carbohydrate to lipid metabolism (37). Ruminants utilize ketone bodies as a source of energy in their peripheral tissues and small intestines, which helps them avoid being deprived of glucose (38). A strong positive correlation has been reported between β-hydroxybutyric acid and C4OH levels, and a moderate correlation between β-hydroxybutyric acid and C2 in the blood (36). The authors also observed that, in accordance with the changes in carnitine, there was an increase in C2 and C4OH with increasing ketosis. These results suggest that short-term dietary BSM supplementation in goats before long-duration transportation may help the animals utilize fat reserves as a source of energy, rather than relying on muscle breakdown, for energy production.

Hexadecenoylcarnitine (C16:1), a long-chain acylcarnitine with unsaturated fatty acid moiety, and hexenoylcarnitine (C6:1), a medium-chain acylcarnitine with unsaturated fatty acid moiety, increased with increasing transportation time in our study. During intense physical stress, acylcarnitines are exported from muscle cells as the carnitine acylation state is higher in cytoplasm than in mitochondria (39). In a moving vehicle, muscles are constantly active in animals to maintain posture and balance. Muscle contraction, which increases glucose and fatty acid oxidation, is the main cause of the increase in long- and medium-chain acylcarnitines in the blood (40). Increased lipolysis and the resulting faster β-oxidation rate than the TCA cycle could be the cause of the increase in long-chain acylcarnitines (41, 42). The ensuing accumulation of fatty acids in the matrix could result in mitochondrial stress and incomplete fatty acid oxidation, leading to acylcarnitines entering circulation (43), as evidenced by the increasing concentrations of long- and medium-chain acylcarnitines with increasing transportation duration.

Sphingolipids are involved in various cellular, developmental, and stress-response processes. Sphingomyelin is produced when a phosphocholine headgroup is transferred from phosphatidylcholine to ceramide, resulting in the production of diacylglycerol (DAG) and sphingomyelin. The most prevalent complex sphingolipids are the sphingomyelin species, which are required for cell survival (44). In our study, TRT had an impact on nine sphingomyelins, with five of these being higher in the BSM group. However, the hydroxylated forms were higher in the C group, and they are known to play a role in cell signaling, including responses to stress and inflammation. A previous study in goats suggested that increased fatty acid metabolism may contribute to higher concentrations of sphingomyelins (21). Ceramide, a substance that can lead to depression, may also increase as plasma sphingosine and sphinganine levels rise (45). In the present study, diet had an impact on 15 phosphatidylcholines, and long-duration transportation stress affected the concentrations of six phosphatidylcholines in goats. The decrease in phosphatidylcholines and their derivatives can be attributed to abnormal metabolism of phospholipids and cell membrane injury, and the increased levels of phosphatidylcholines, sphingomyelins, and their derivatives can be associated with increased fatty acid metabolism due to transportation stress. However, because there were changes in the concentrations of phosphatidylcholines and their derivatives in both TRT groups, the effect of the BSM diet on goats is not clear under the conditions of the experiment and requires future investigation.

Carnosine levels were higher in the C group compared to the BSM group. Carnosine is useful in preventing the damage caused by stress in animals. It is formed from the binding of the amino acids, alanine and histidine, which present a binding site for glucose. Carnosine has been reported to improve glucose metabolism in stressed animals. The increased carnosine concentration in the C group was likely because these goats experienced higher stress levels and thus required an elevated carnosine-mediated coping mechanism. In our study, creatinine decreased during the initial 4 h of transportation and then gradually increased with increasing transportation time. Creatinine is a non-protein nitrogenous compound that is produced by the breakdown of creatine in muscle. Creatine conversion to phosphocreatine is catalyzed by creatine kinase, with spontaneous formation of creatinine during the reaction (46). Muscle activity, heat stress, dehydration, and glomerular filtration rate have all been reported to increase blood creatinine concentrations. In the present study, creatinine levels began to increase after 4 h of transportation, while stress levels decreased based on epinephrine concentrations. Dehydration, rather than stress response, likely caused the increase in creatinine after 4 h, as the goats had access to ad libitum water until they were loaded onto the trailers, and dehydration did not occur until after the initial few hours. Previous studies in small ruminants have shown increases in creatinine concentrations after 20 h of transportation (47), severe water restriction (48), or heat stress (49).

Black cumin has been reported to reduce blood glucose by lowering hepatic gluconeogenesis (50). In our study, gluconeogenic metabolites (propionic acid and methylmalonic acid) and glucose were found to be lower in the BSM group than in the C group. The lower glucose and amino acid concentrations in the BSM group compared to the C group may be due to lower stress, proper glucose uptake by the muscle cells, lower hepatic gluconeogenesis, and lower protein degradation. Methylmalonic acid is produced in the body when it is necessary for energy production by breaking down proteins (51). The higher unsaturated acylcarnitines and low amino acid concentrations in the BSM group may also indicate the use of fat reserves for energy, instead of muscle breakdown or protein catabolism. As BSM lowers hepatic gluconeogenesis, the amino acids are likely not catabolized into TCA cycle metabolites (α-ketoglutaric acid, succinic acid, and fumaric acid), as noticed in the present study. The higher isobutyric acid concentrations in the BSM group further confirm the predominance of lipid metabolism in the BSM group.

Pathway analysis results confirm the paper’s central finding: the two groups of goats used different energy-sourcing strategies during transportation stress. The C group had significantly higher concentrations of numerous amino acids and TCA cycle intermediates. This is interpreted as the C group breaking down muscle protein for energy. The pathway analysis strongly corroborates this by flagging these exact amino acid and energy pathways as the primary areas of metabolic difference between the BSM and C groups. Since the metabolomics analysis revealed prominent effects mainly on energy-sourcing strategies in the two groups of goats, the metabolomics results could not be related to the antioxidant and immune status variables determined in this study. The catecholamine analysis to assess stress levels during transportation revealed significant effects on tyramine concentrations, which were attributed to dietary treatment. While the effects of stress on antioxidant and immune capacities in animals are well established, the differences in antioxidant and immune capacity indicators between the two groups of goats after exposure to intense stress were not immediately evident in the present experiment. It can be speculated that changes in the markers related to these variables may manifest 24–48 h after transportation under the conditions of this study.

Limitations of this study were that it was conducted on a commercial farm under routine practices typically followed on that farm. Therefore, replications of corrals could not be established, nor could the feed intake by individual animals during the three-week treatment period be recorded. Therefore, dietary effects should be interpreted with caution. Additionally, ether extract, crude protein, and thymoquinone contents in the BSM batch used in this study were not available. Since the primary clients of this farm were other meat goat farmers nationwide and small ruminant researchers at universities, the goats were fed a concentrate diet exclusively, particularly during the summer. This is not representative of meat goat farms in the southeastern US, where goats are typically raised on pastures with concentrate and hay supplements and sent to processing plants either directly or through livestock auctions.

## Conclusion

Black seed meal supplementation did not differentially affect physiological responses, except tyramine concentrations. The lower glucose and TCA cycle metabolites in the BSM group suggest an ability of BSM to lower stress, enhance proper glucose uptake by the muscle cells, lower hepatic gluconeogenesis, and lower protein degradation. Metabolomic profiles suggest a shift toward lipid utilization when goats were fed BSM for a brief period before long-duration transportation. However, physiological and immune status indicators could not be related to this conclusion. Despite higher concentrations of amino acids that can help enhance immune function, energy balance, and anti-inflammatory activity, the antioxidant and immune status indicators determined did not support this conclusion. Further studies are required under controlled conditions that enable monitoring of feed intake and weight gain at different levels of BSM in the diet. The data will help us better understand the optimal level of inclusion of this product in the diet, which has a positive impact on animal welfare, stress resistance, and productivity.

## Data Availability

The original contributions presented in the study are included in the article/Supplementary material, further inquiries can be directed to the corresponding author.
