# Supplementary material for: On-farm dietary supplementation of black seed (Nigella sativa) meal in goats: effects on physiological and metabolomic responses during transportation
Source: Front Vet Sci. 2026 Jan 22;12:1721007. doi: 10.3389/fvets.2025.1721007 (PMC12872573; doi:10.3389/fvets.2025.1721007)
Supplement: Supplementary file 1 [file Data_Sheet_1.pdf]

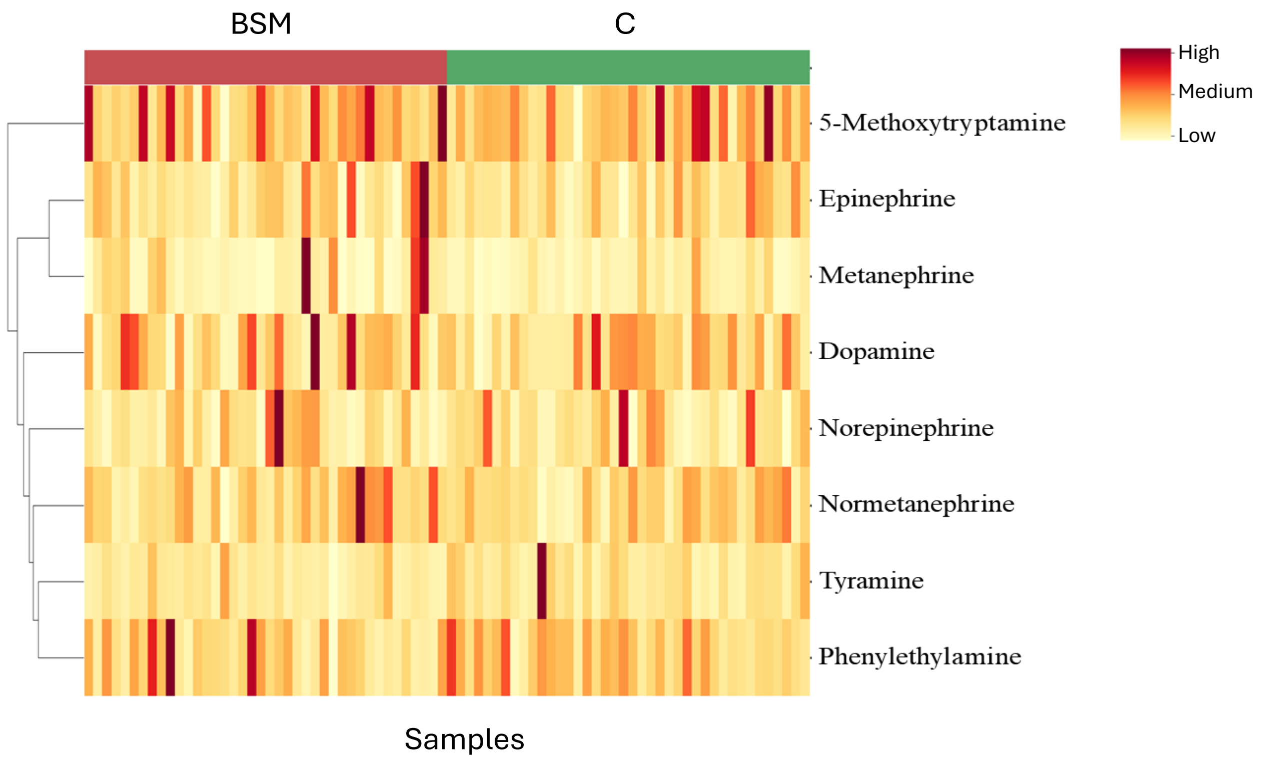

Supplementary Figure 1. Heat map showing the concentrations of catecholamines and their derivatives affected by TRT, created using normalized concentration ranges (from 0 to 1). High, medium, and low points on the color bar correspond to 0.95, 0.5, and 0.05, respectively.

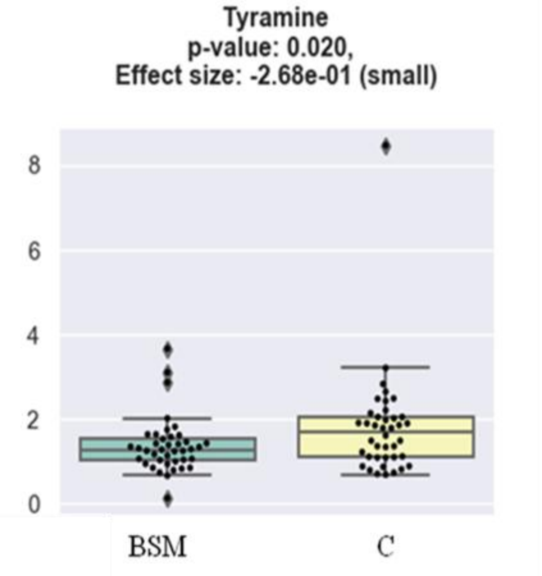

Supplementary Figure 2. Box plots of catecholamines and other biogenic amines significantly ($P < 0.05$) affected by treatment (BSM = Black seed meal; C = Control) in goats.

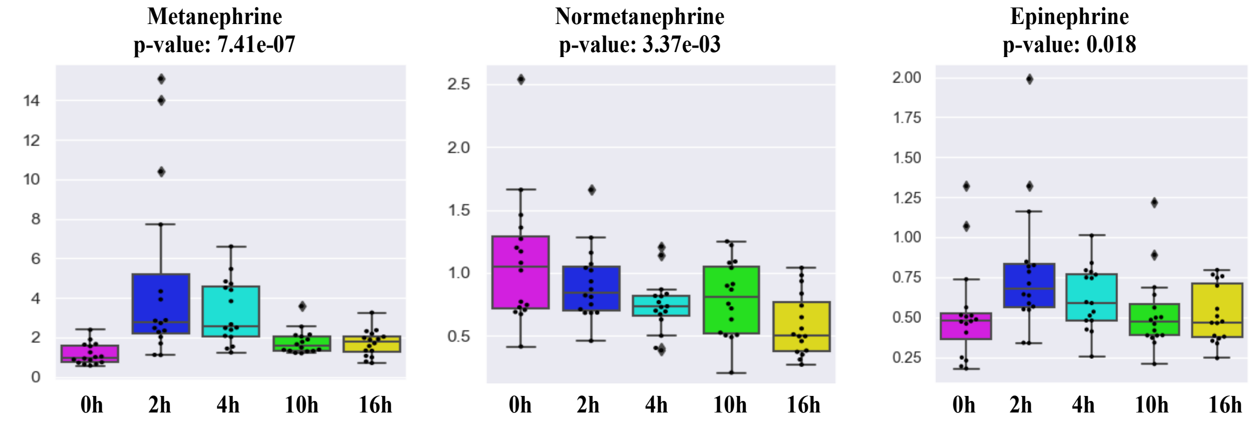

Supplementary Figure 3. Box plots of catecholamines (nM) significantly ($P < 0.05$) affected by transportation time.

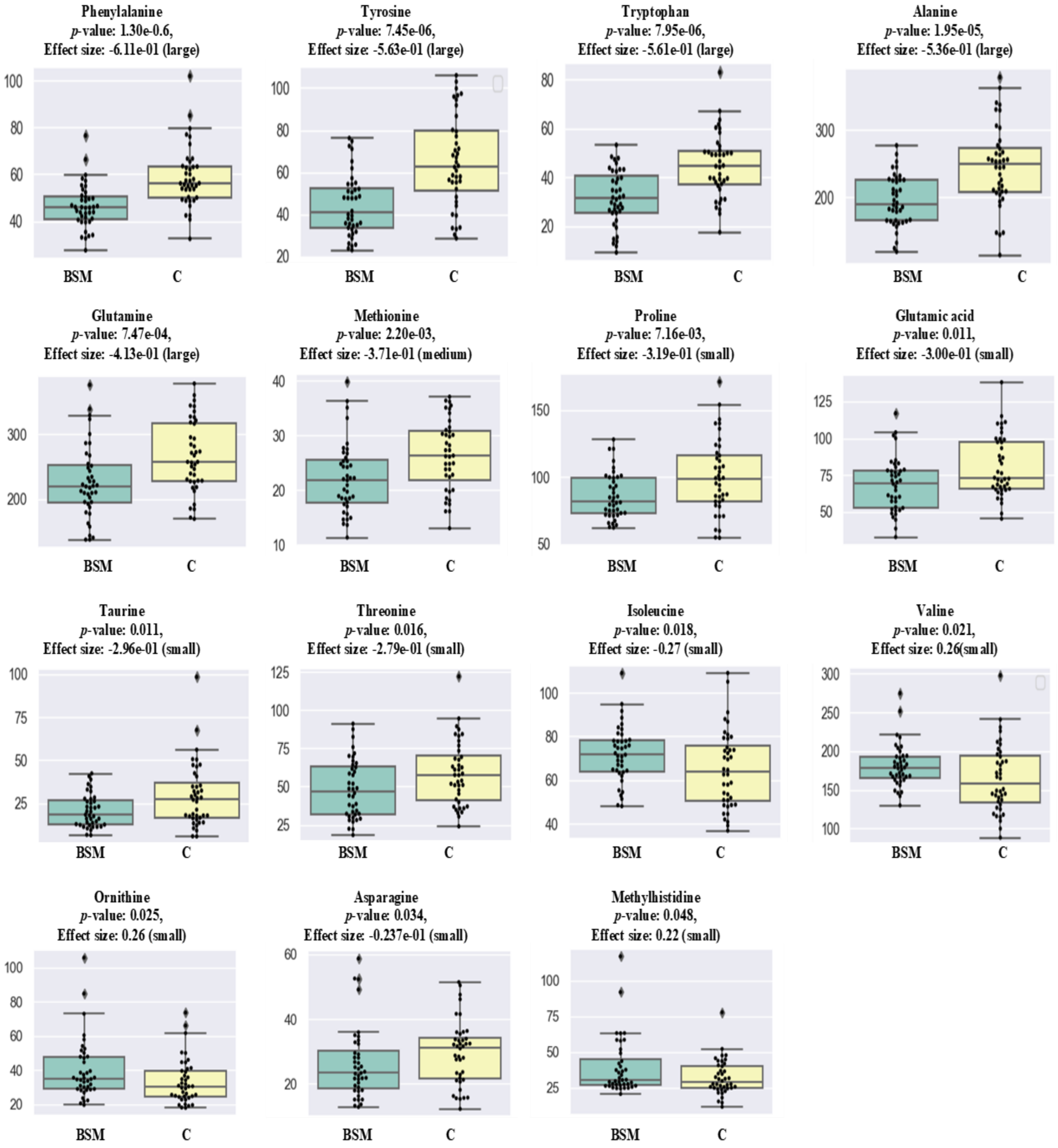

Supplementary Figure 4. Box plots of amino acids significantly (P < 0.05) affected by treatment (BSM = Black seed meal; C = Control) in goats.

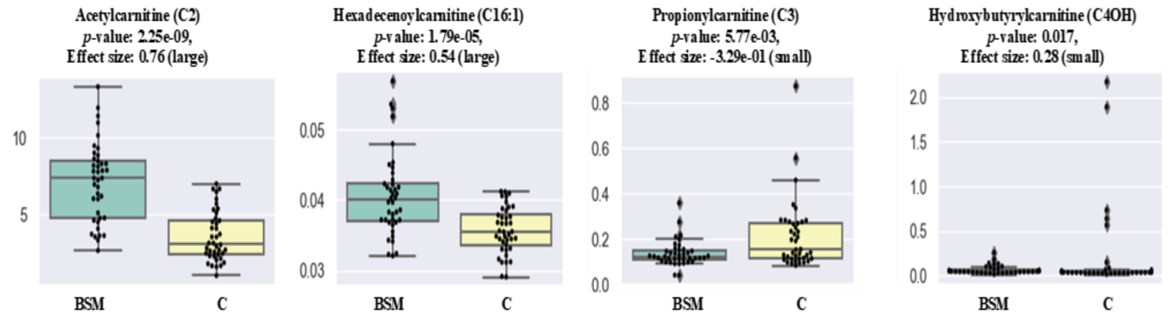

Supplementary Figure 5. Box plots of acylcarnitines significantly (P < 0.05) affected by treatment (BSM = Black seed meal; C = Control) in goats.

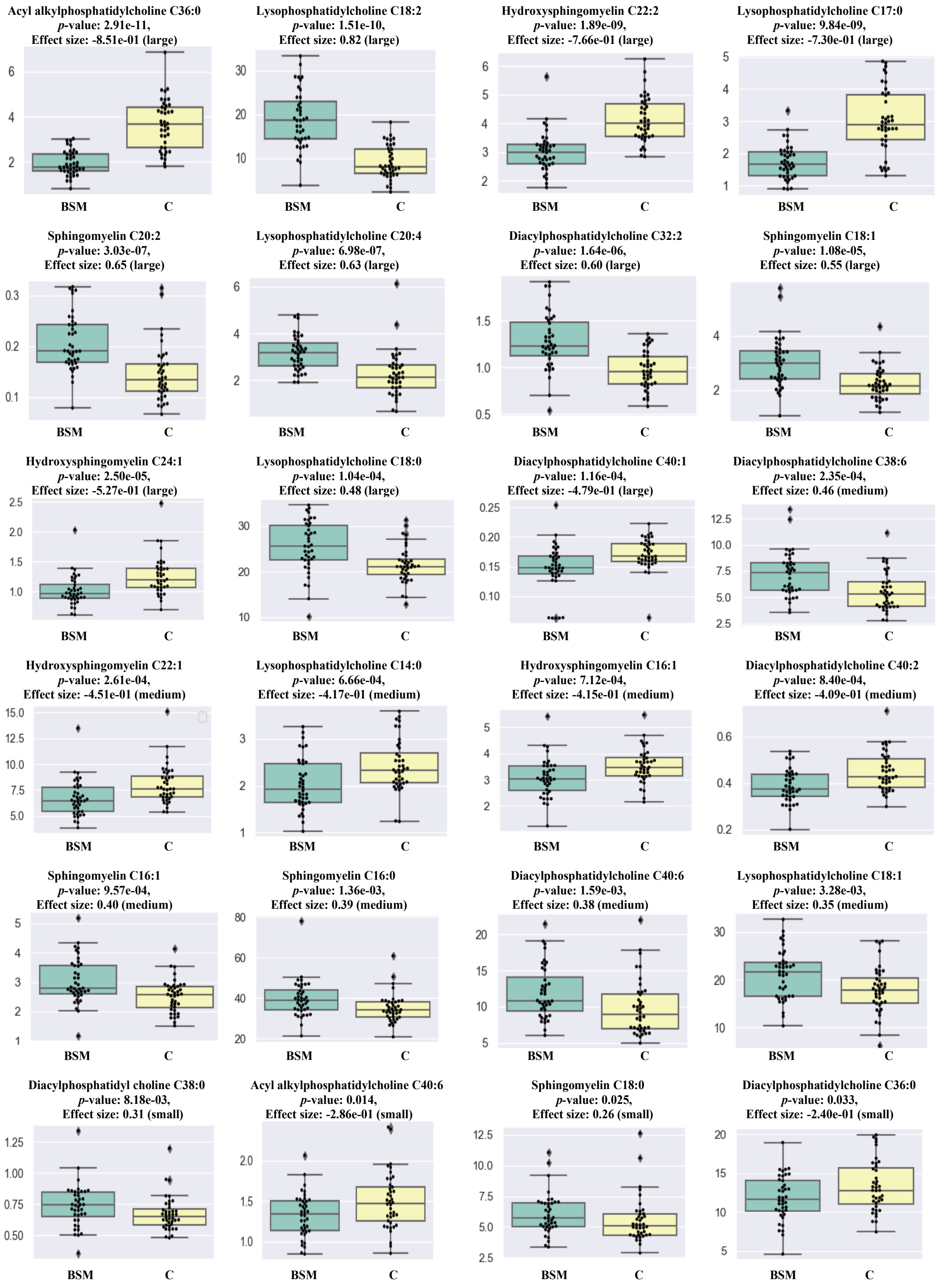

Supplementary Figure 6. Box plots of phosphatidylcholines and sphingomyelins significantly (P < 0.05) affected by treatment (BSM = Black seed meal; C = Control) in goats.

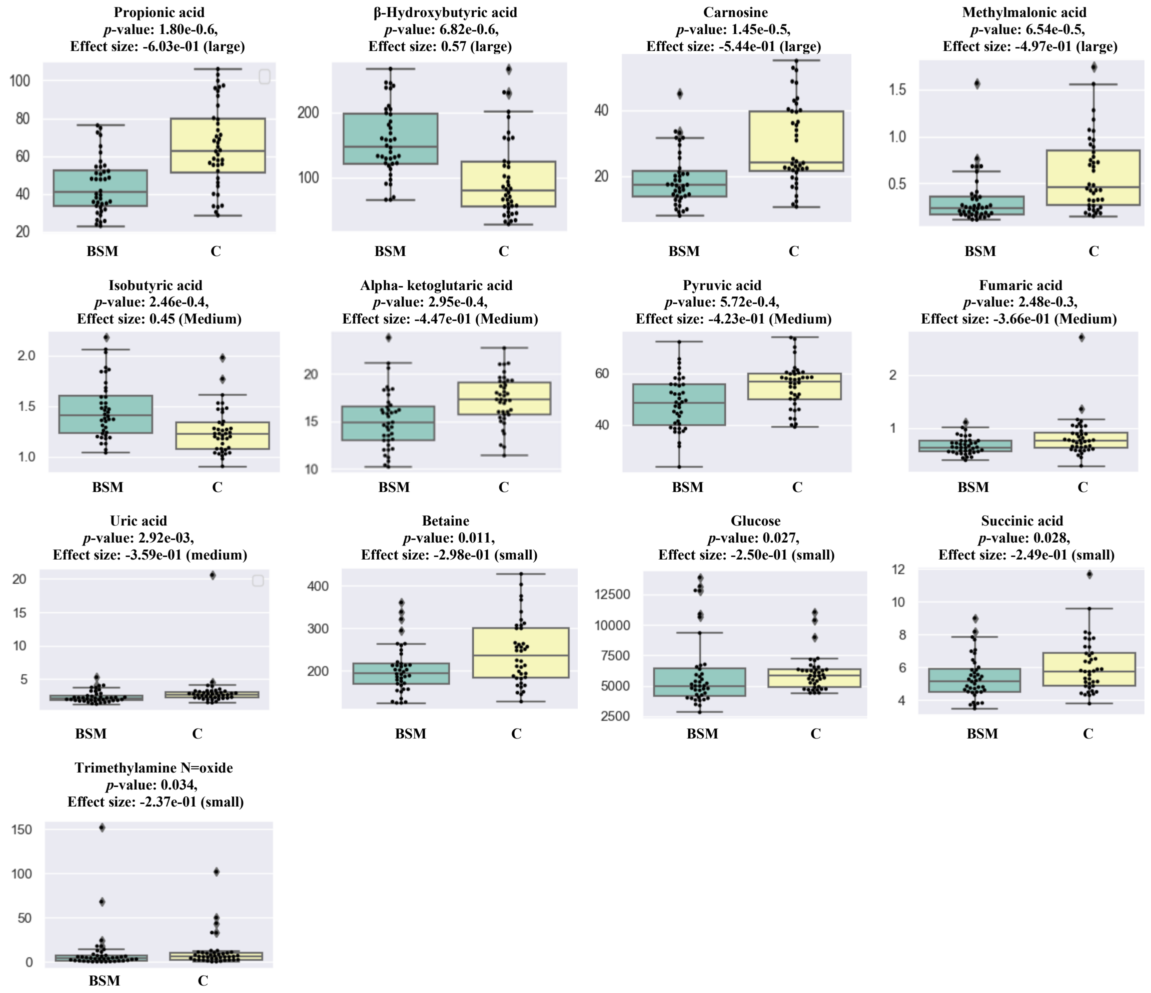

Supplementary Figure 7. Box plots of metabolites significantly (P < 0.05) affected by treatment (BSM = Black seed meal; C = Control) in goats.

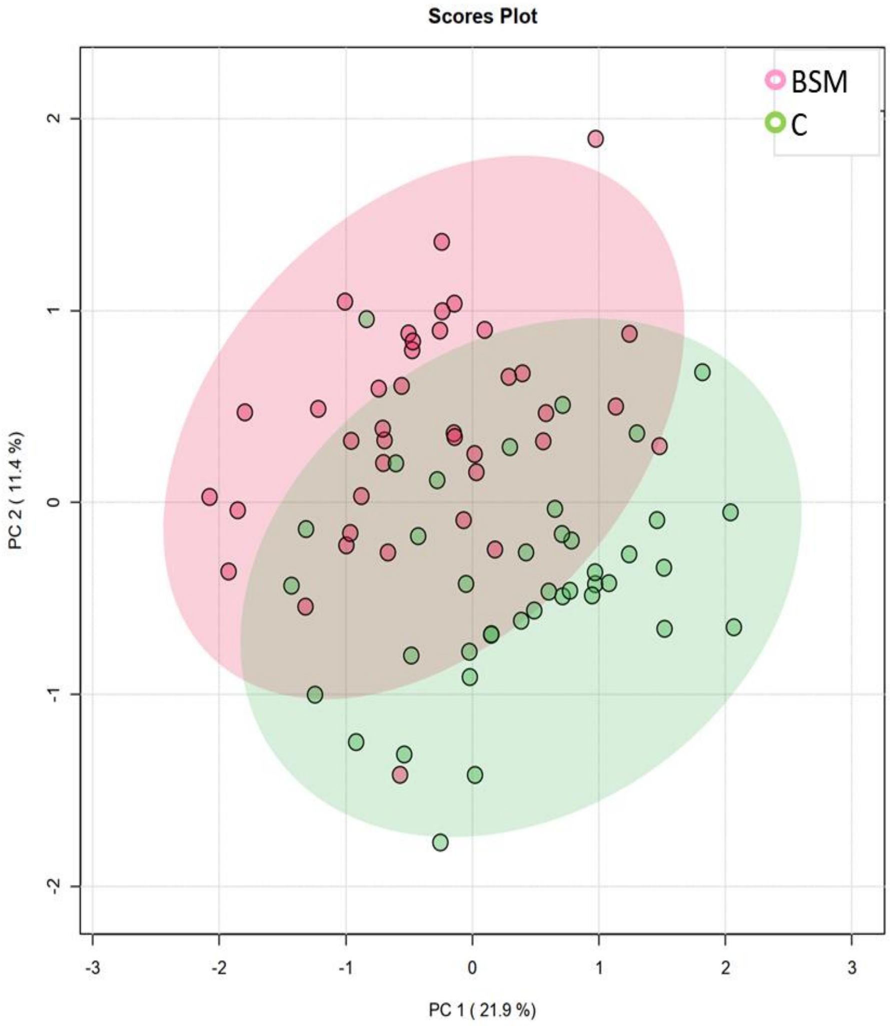

Supplementary Figure 8. PCA plot of principal components 1 and 2 for treatment classes (BSM = Black seed meal, C = Control; $P < 0.05$) in goats.

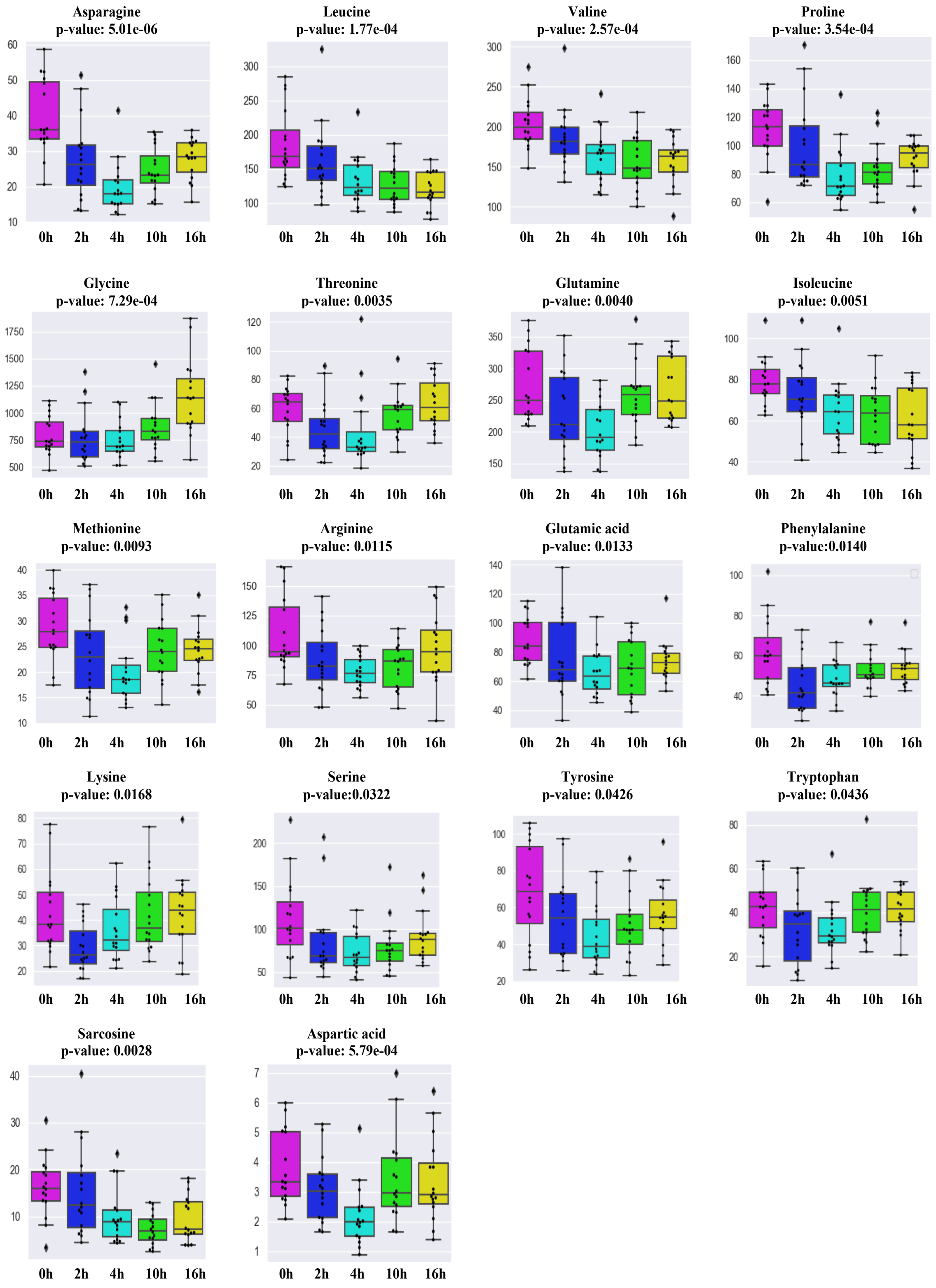

Supplementary Figure 9 Box plots of amino acids significantly ($P < 0.05$) affected by transportation time (0 h, 2 h, 4 h, 10 h, and 16 h) in goats.

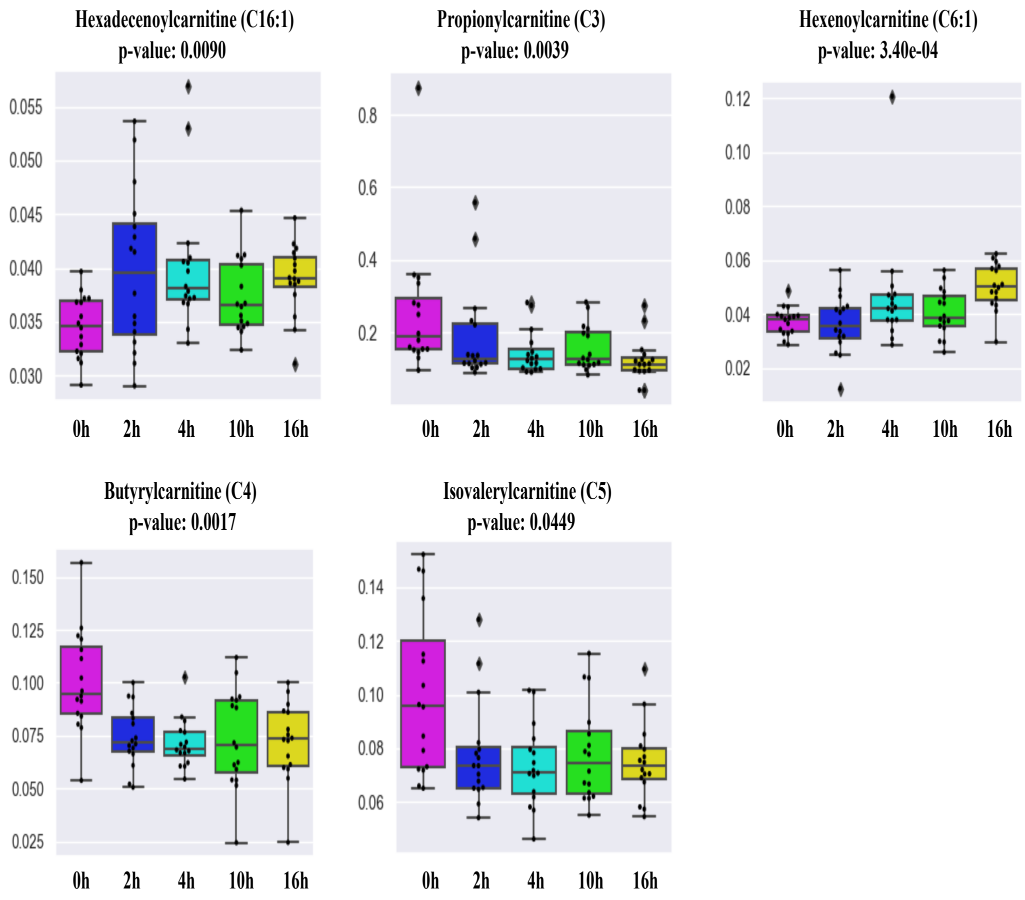

Supplementary Figure 10. Box plots of acylcarnitines significantly ($P < 0.05$) affected by transportation time (0 h, 2 h, 4 h, 10 h, and 16 h) in goats.

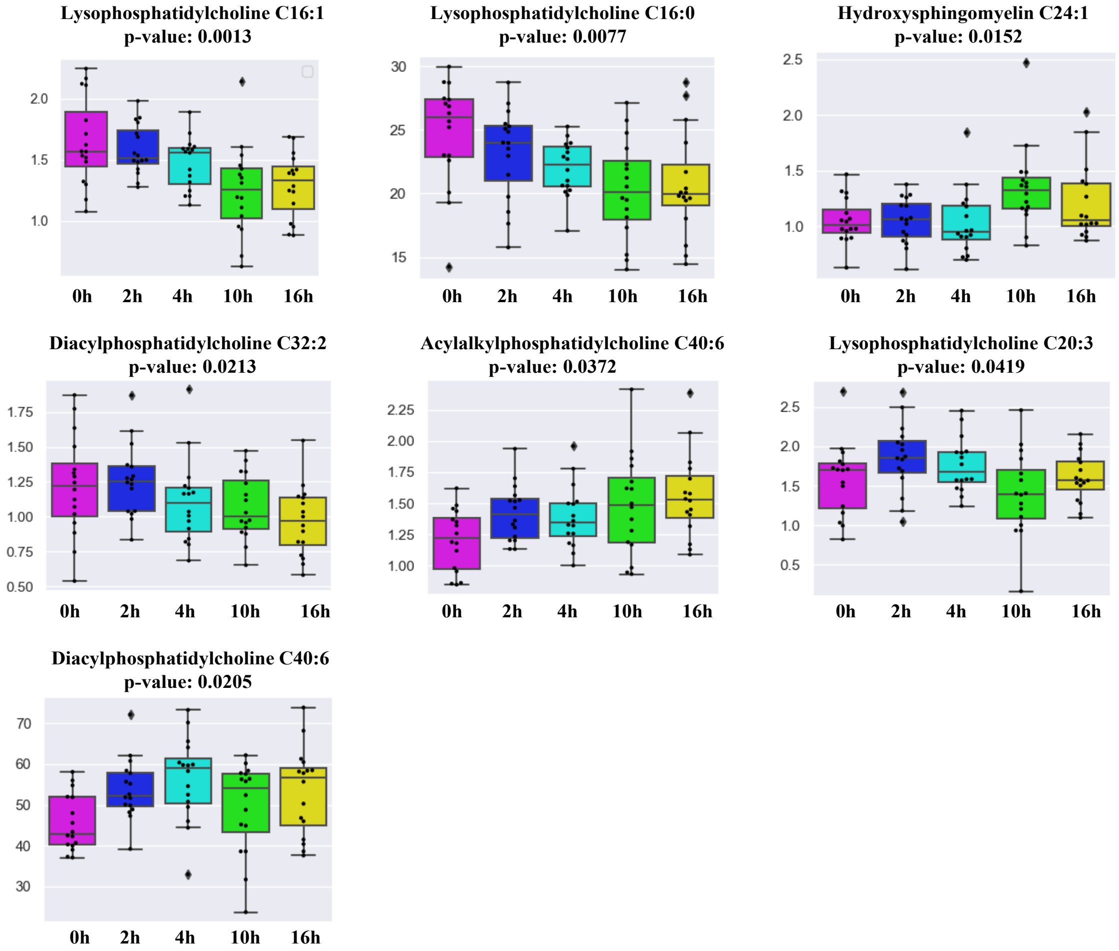

Supplementary Figure 11. Box plots of phosphatidylcholines and sphingomyelins significantly (P < 0.05) affected by transportation time (0 h, 2 h, 4 h, 10 h, and 16 h) in goats.

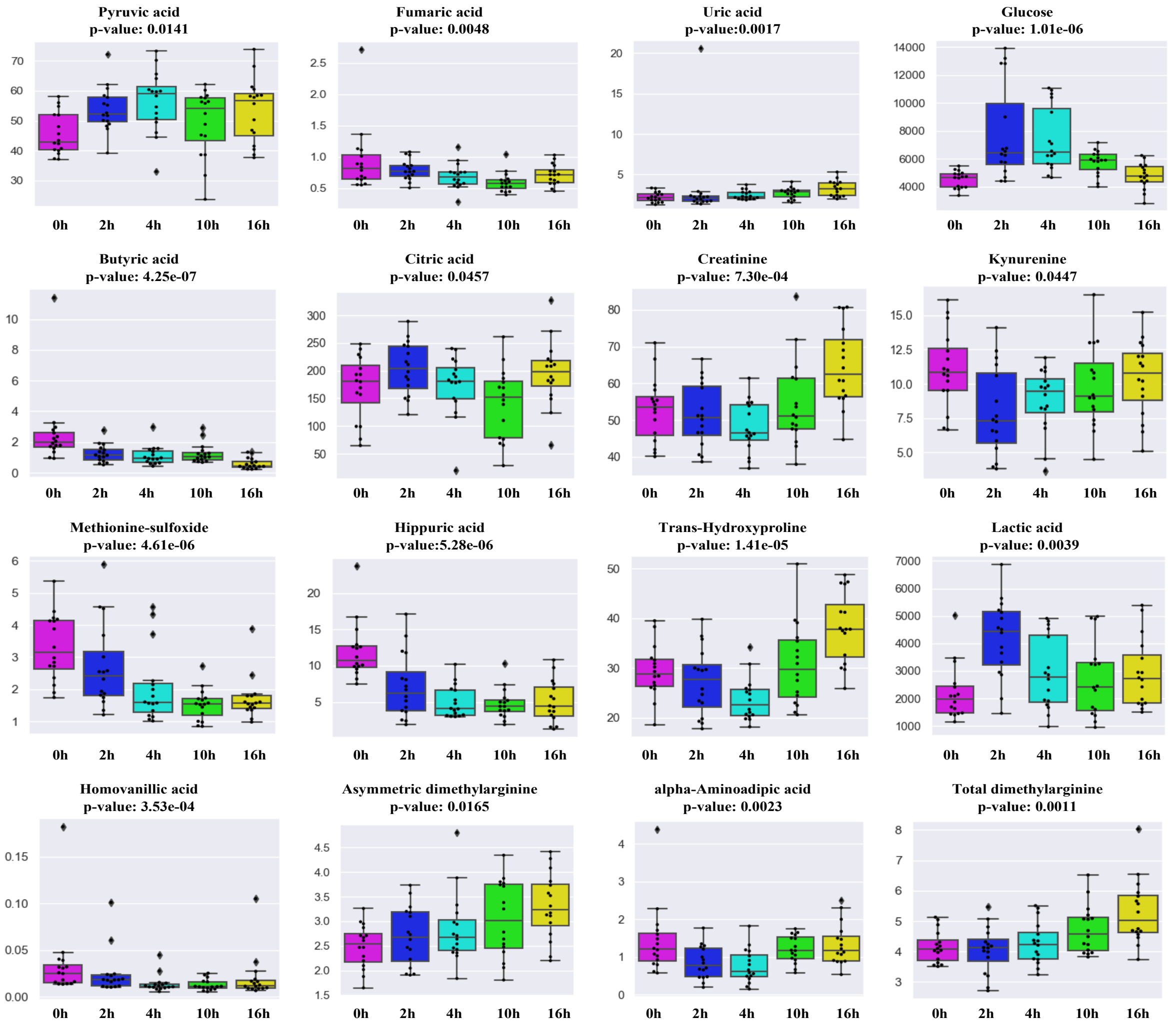

Supplementary Figure 12. Box plots of metabolites significantly ($P < 0.05$) affected by transportation time (0 h, 2 h, 4 h, 10 h, and 16 h) in goats.

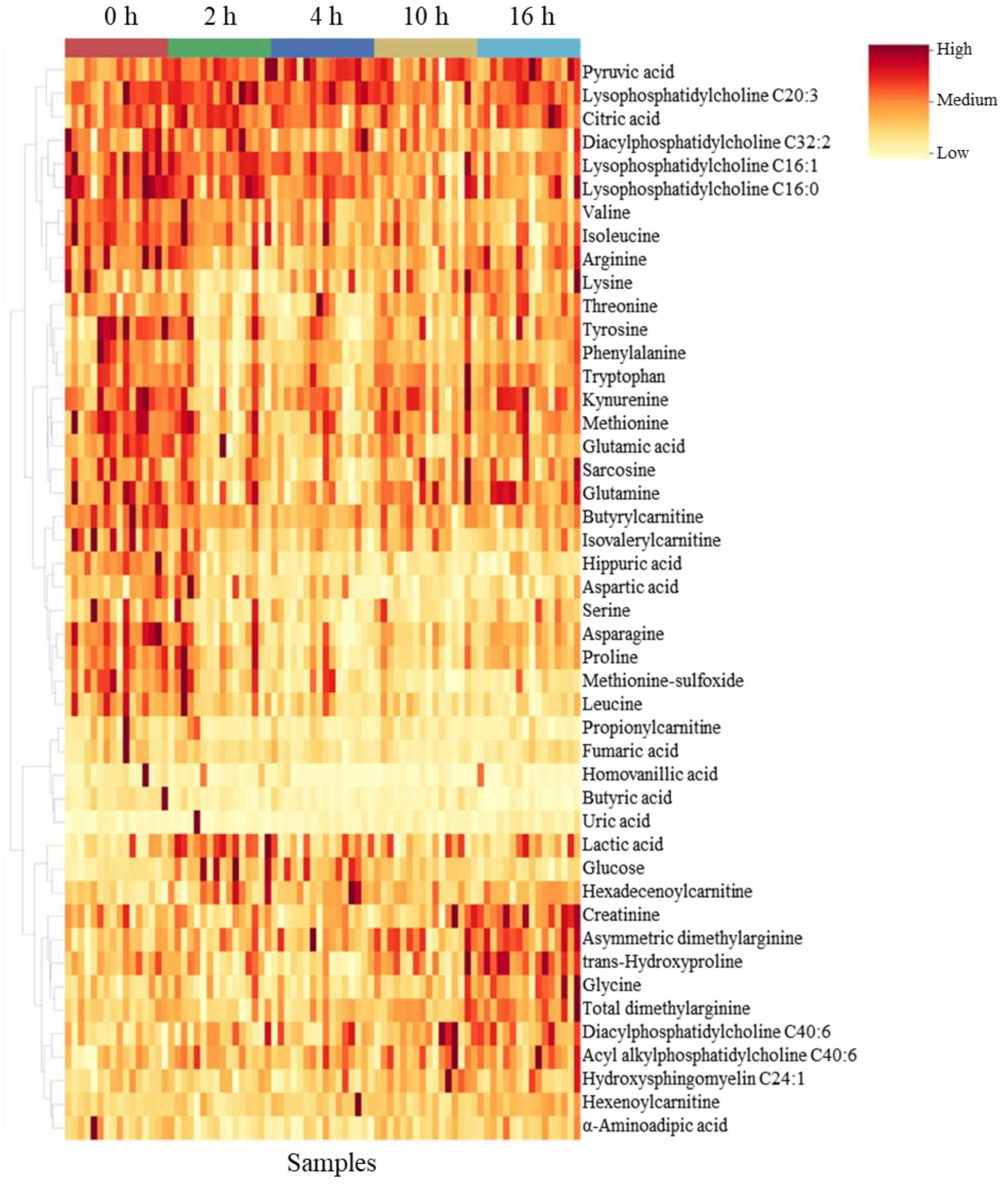

Supplementary Figure 13. Heatmap of significant plasma metabolites clustered by transportation time (0 h, 2 h, 4 h, 10 h, 16 h; $P < 0.05$) in goats.

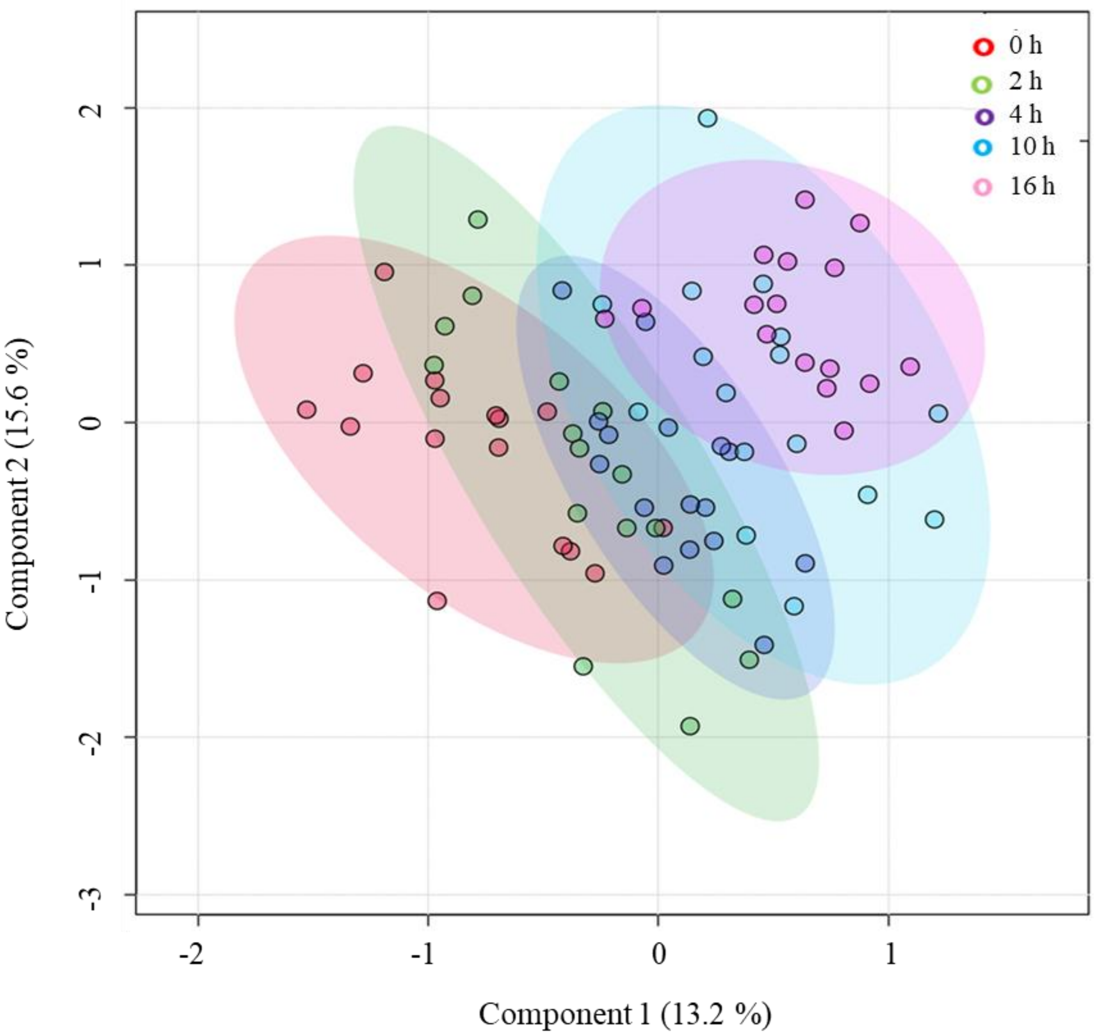

Supplementary Figure 14. PLS-DA plot of principal components 1 and 2 for transportation time (0 h, 2 h, 4 h, 10 h, and 16 h) for the metabolites in goats.

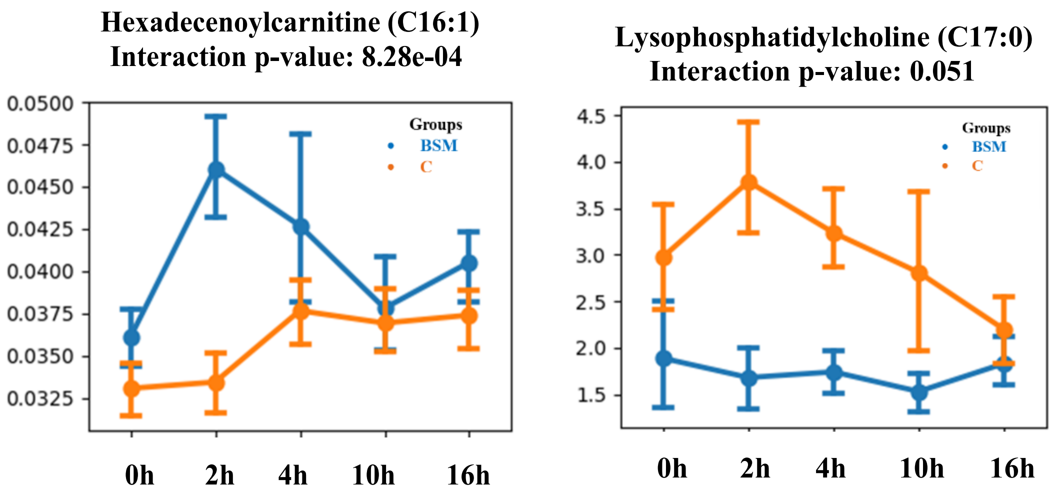

Supplementary Figure 15. Graph showing the treatment (BSM = Black seed meal; C = Control) and transportation time (0 h, 2 h, 4 h, 10 h, and 16 h) interaction effect on hexadecenoylcarnitine and lysophosphatidylcholine in goats.

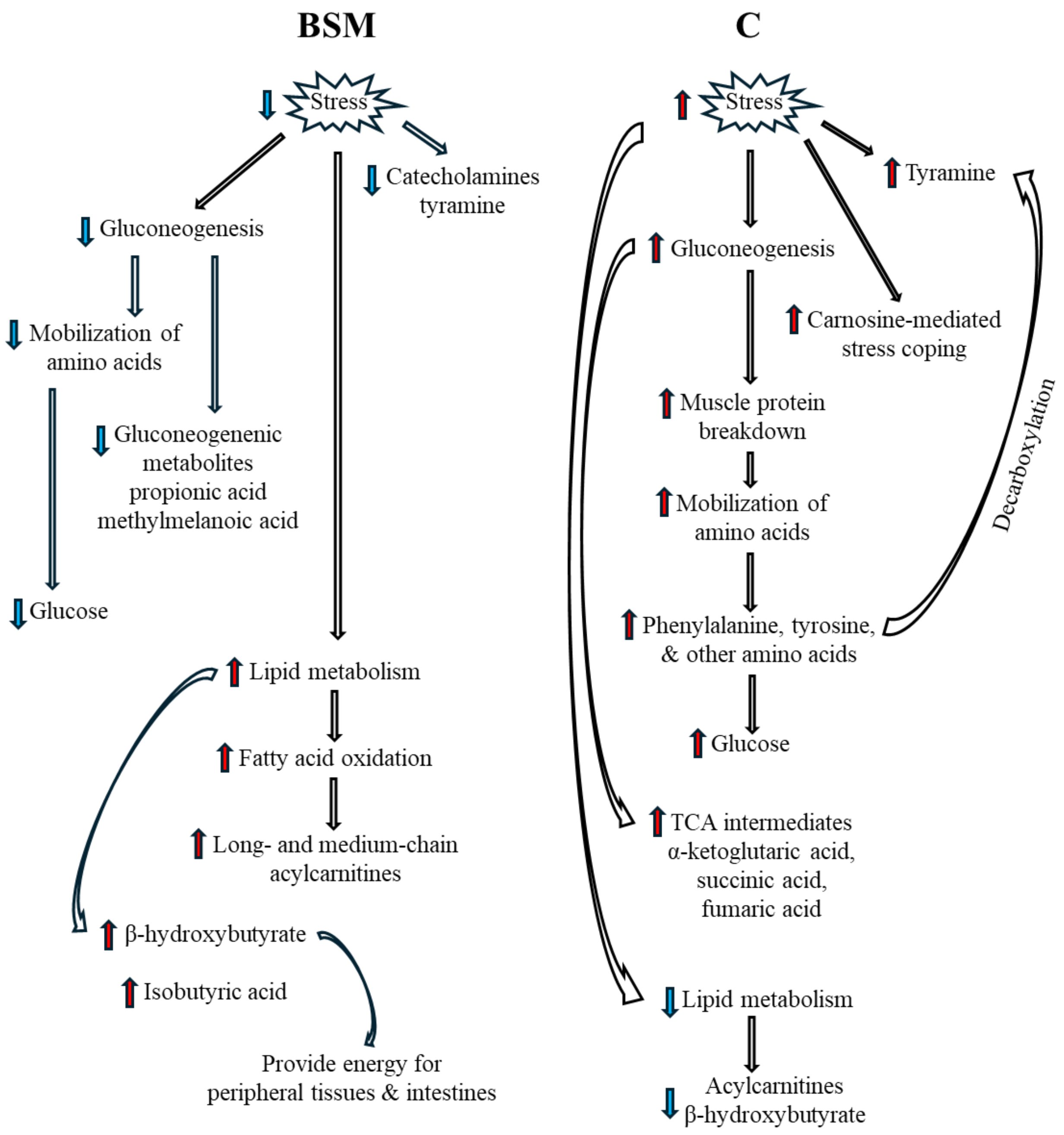

Supplementary Figure 16. Mechanistic diagram showing the differences in energy-sourcing strategies between the two treatment groups (BSM = Black seed meal; C = Control) based on metabolomics analysis.
